# A Natural Approach to Improving Berry Quality in Table Grapes: Inactivated Yeast Extracts

**DOI:** 10.3390/molecules31193362

**Published:** 2026-09-22

**Authors:** Muhammet Dagla, Sevil Canturk, Erdal Agcam, Asiye Akyıldız

**Affiliations:** 1 Institute of Natural and Applied Sciences, Çukurova University, Adana 01330, Türkiye; muhammetdagla01@gmail.com; 2 Department of Horticulture, Faculty of Agriculture, Çukurova University, Adana 01330, Türkiye; 3 Department of Food Engineering, Faculty of Engineering, Çukurova University, Adana 01330, Türkiye; eagcam@cu.edu.tr (E.A.); asiye1@cu.edu.tr (A.A.)

**Keywords:** *Saccharomyces cerevisiae*, yeast extract, table grape, quality, skin color, yield, phenolic compounds

## Abstract

Inactivated yeast extracts derived from *S. cerevisiae* have recently gained attention as natu-ral and environmentally sustainable elicitors. In this study, the effect of foliar yeast treat-ment on anthocyanin and phenol profiles, antioxidant activity, skin color, and table grape quality characteristics of *V. vinifera* L. cv ‘Early Cardinal’ and ‘Trakya Ilkeren’ was inves-tigated. Yeast extract (Y) was applied at doses of 0.5 kg ha^−1^ (Y1), 1 kg ha^−1^ (Y2), 2 kg ha^−1^ (Y3), and 4 kg ha^−1^ (Y4). In Early Cardinal, all yeast doses improved cluster and berry characteristics. In Trakya Ilkeren, cluster weight peaked in the Y3 treatment. No clear trend was observed in sugars and organic acids in either cultivar. Only small variations were observed in total anthocyanins and berry skin color. In Early Cardinal, Y2, Y3, and Y4 treatments significantly increased malvidin*-3-O-*glucoside, petunidin*-3-O-*glucoside, and peonidin*-3-O-*glucoside. In Trakya Ilkeren, the anthocyanin profile remained stable. Total phenol content was measured by the Folin–Ciocalteu assay and expressed as gallic acid equivalents; it increased in all treatments, although no consistent increase in individual phenols was observed. The highest antioxidant activity (DPPH (1,1-diphenyl-2-picrylhydrazyl) and FRAP (ferric reducing antioxidant power)) was detected in Early Cardinal with Y3 and Y4 doses, and in Trakya Ilkeren with Y1 and Y3 treatments. The study provided preliminary insights into improving the quality and nutritional value of table grapes under warm conditions through a natural component of vineyard ecosystems.

## 1. Introduction

Obtaining the desired grape quality depends on achieving two critical targets: “technological maturity”, which is defined as the accumulation of sugars and acids in the berries, and “phenolic maturity”, which refers to the optimum concentration of phenolic compounds. Increasing global temperatures and drought over the last 20–30 years have seriously affected grapevine phenology, causing véraison to occur earlier than expected [1,2]. As a result of this accelerated ripening, phenolic and aromatic structures in table grapes remain poor, and imperfect skin color can be observed [3,4]. Normally, anthocyanin biosynthesis begins during véraison in pink, red, and black-skinned grape cultivars, and anthocyanin continues to accumulate in berry skin layers until maturity. However, studies reported the inhibition and degradation of anthocyanin biosynthesis in grape skins at temperatures above 30 °C [5,6,7,8]. Inadequate and inhomogeneous skin color at harvest is a significant problem, particularly reducing the market value of pink and red table grapes.

Several cultural practices in viticulture, such as pruning and training systems [9], cluster thinning [10], girdling [11], and leaf removal [12], mitigate the negative effects of high temperatures on berry skin color. While these methods improve skin color by promoting the biosynthesis of anthocyanins and phenolic compounds in grape berries, they can also negatively affect berries, such as reducing berry size and, in some cases, causing sunburn [13]. Therefore, in recent years, using “elicitors”, which stimulate plant defense mechanisms, has become increasingly common as an alternative to cultural practices. Elicitors are molecules of abiotic and biotic origin that can stimulate plant stress responses and trigger the biosynthesis or the accumulation of secondary compounds that play a crucial role in adaptation to stressful conditions [14,15]. Eco-friendly applications have gained increasing interest in recent years, and, consequently, the elicitor potential of yeasts in grapevine and various plant species has begun to be explored. Although many yeast species live in grapevine organs, *Saccharomyces cerevisiae* is critical in grape berry development and the technological processing of grapes. Yeast cell membranes are composed of mannoproteins, *β-*glucans, and chitin, while yeast plasma membranes are lipids, sterols, and proteins. Many of these components stimulate various plant defense mechanisms [14,16]. Although yeasts are widely used in in vitro studies because of these properties [17,18,19], their use as in vivo elicitors is a comparatively new field of study. Inactivated yeast extracts derived from *Saccharomyces cerevisiae*, a natural component of vineyard ecosystems, are currently considered among the most natural and environmentally sustainable treatments [13]. Previous studies have focused on wine grapes because yeasts play a key role in wine’s technological and sensory qualities. Several studies reported that the interaction of mannoproteins in yeasts with anthocyanins and tannins ensures berry skin color stability and controls astringency in red wines [20,21]. Studies on many world-renowned wine grape cultivars have shown that foliar yeast treatments can improve phenolic and aromatic profiles and enhance varietal expression by influencing plant secondary metabolism without altering original sugar and acid levels [22,23,24,25]. Assessing the impact of yeasts on table grape quality is still in its infancy. The first studies on table grapes were Crupi et al. [13] and Petoumenou and Patris [26], on the cultivars that exhibit poor skin color in warm years. Visual perception and nutritional value remain the most critical parts of table grape quality assessment. Intense and uniform berry skin color not only ensures visual appeal but also provides a rich source of secondary metabolites known to have positive health effects. However, because table grapes are consumed fresh, natural methods should improve their physical and chemical quality. Therefore, yeasts naturally present in grapevines could be considered a sustainable way to improve skin color and chemical quality of table grapes, especially in warm regions with Mediterranean climates, where the temperature difference between day and night is low.

This study aimed to evaluate the enhancing effect of foliar-applied yeast (*Saccharomyces cerevisiae*) extracts on berry skin color, anthocyanin content, yield, and overall quality parameters of early-ripening table grape cultivars ‘Early Cardinal’ and ‘Trakya Ilkeren’ under Mediterranean climate conditions. To the best of our knowledge, this is the first study to test four yeast doses to determine the appropriate dose for improving quality under warm conditions.

## 2. Results and Discussion

### 2.1. Influences on Yield, Cluster, and Berry Characteristics

Table 1 shows the effects of yeast treatment on yield, cluster, and berry characteristics in the studied cultivars. In Early Cardinal, vine yield ranged from 10.46 kg (Y4) to 7.68 kg (Y1) and did not differ significantly across the treatments. However, the highest yield was obtained from the highest dose (Y4). For cluster weight, the highest values were observed for the Y1 (331.27 g) and Y2 (314.47 g) treatments, which were also significantly higher than the others. While the Y2 and Y4 doses significantly increased cluster length, all the treatments increased cluster width. Although all treatments improved berry weight, length, and width, the Y1 dose yielded the highest values. In Trakya Ilkeren, vine yield ranged from 13.46 kg (Y4) to 10.13 kg (Control). While all treatments produced a slight increase in vine yield, the increase was not statistically significant. However, cluster weight showed positive effects. All treatments significantly increased cluster weight compared with the control, but the largest increases were observed with the Y3 (415.43 g) and Y2 (397.01 g) doses. Small increases in cluster length were not statistically significant, whereas the 2 kg ha^−1^ dose (Y3) significantly increased cluster width. Even though the treatments also produced small increases in berry weight, length, and width, the effects did not reach statistical significance.

Similar to our findings, previous studies on grapevines generally indicate that yeast extracts have limited impact on yield components. Many authors have reported that yeast treatment applied at various concentrations and under different ecological conditions has no significant effect on yield and cluster weight across grape cultivars [25,27]. Conversely, Del Zozzo et al. [28] reported that proline-containing yeast extracts enhanced yield, cluster and berry weight, and cluster compactness in ‘Barbera’ cultivar. In the present study, we obtained similar results for cv ‘Early Cardinal’. All the yeast doses led to varying degrees of improvement in cluster and berry characteristics in this cultivar. However, in ‘Trakya Ilkeren’, significant effects were present only for cluster weight and width. Similar inconsistencies exist for physical berry characteristics. For instance, Portu et al. [22], Kogkou et al. [29], and Duilio and Battista [25] reported no significant impact of yeast treatment on berry weight in ‘Tempranillo’, ‘Agiorgitiko’, and ‘Gewürztraminer’. The physical characteristics of clusters and berries are prominent quality parameters for table grapes. One study on table grapes was conducted by Crupi et al. [13] on ‘Red Globe’, ‘Scarlotta Seedless’, and ‘Crimson Seedless’ cultivars. They reported that foliar yeast treatment at 1 kg ha^−1^ had negligible effects on berry size and textural properties. Consistently, the 1 kg ha^−1^ dose had a minor effect on berry traits of ‘Trakya İlkeren’ in the present study.

In contrast, Petoumenou and Patris [26] reported that foliar yeast treatment of 1.5 kg L^−1^ significantly increased berry weight and size in ‘Crimson Seedless’ cultivar compared to the control group. In this study, the 1 kg ha^−1^ dose also positively affected berry weight and size in ‘Early Cardinal’. These findings suggest that the two cultivars responded differently to the yeast treatments; however, since the cultivars were analyzed separately and no cultivar × treatment interaction was tested, this difference is described as a qualitative observation only. While no single yeast dose stood out across either cultivar in our study, the favorable effects on cluster and berry characteristics were more pronounced in ‘Early Cardinal’. In contrast, although cluster weight and width increased in ‘Trakya Ilkeren’, the effects on berry traits remained much more limited.

### 2.2. Influences on Technological Ripeness, Sugars and Acids

Yeast treatments altered the sugar and organic acid content of the must in both cultivars; however, no clear trend existed (Table 2). In Early Cardinal, despite minor fluctuations in TSS (total soluble solids) values among treatments, no significant differences were observed compared with the Control. TSS values ranged from 16 to 17 °Brix. The Y1 and Y4 doses slightly increased pH, whereas the Y4 dose significantly decreased titratable acidity. The maturity index also increased at the 4 kg ha^−1^ dose (Y4), reflecting the changes in TSS and acidity. Furthermore, glucose and fructose concentrations decreased at Y3 compared to the control; conversely, sucrose content increased significantly across all doses except for the 2 kg ha^−1^ (Y3) treatment. For organic acid profiles, tartaric acid peaked at the Y3 dose, whereas the Y1 dose dropped significantly compared to the control. Malic acid showed no significant differences among treatments, while citric acid decreased notably at the Y1 and Y4 doses.

For minor organic acids, ascorbic acid increased significantly at the Y3 dose, and fumaric acid at Y4. Oxalic acid levels were unaffected by the treatments. In Trakya Ilkeren, treatments did not affect TSS or pH; in contrast, titratable acidity slightly decreased at the Y1, Y2, and Y3 doses. The highest maturity index was achieved at Y2 because acidity decreased. Although glucose and fructose concentrations fluctuated slightly, these changes were not significant; however, the Y1, Y3, and Y4 doses noticeably reduced the sucrose content. The treatments strongly influenced the organic acid profiles of this cultivar. The Y2 dose generally reduced tartaric, malic, and citric acids. In contrast, the Y4 dose reduced citric acid, while increasing tartaric and malic acid levels compared to the control. Among the minor organic acids, ascorbic acid was unaffected by the treatments, whereas oxalic and fumaric acids increased or decreased significantly depending on the doses.

Numerous previous studies have reported that foliar yeast treatments do not significantly alter must components such as TSS, pH level, and titratable acidity [13,22,24,25,27]. Similarly, Rodrigues et al. [30] reported that yeast treatment at 4 kg ha^−1^ had no significant effect on the total sugar or total acidity of ‘Sauvignon Blanc’. Although the primary goal of yeast treatments in grapevines is not to alter the balance of taste, some studies have noted minor fluctuations in sugar and organic acid profiles. For instance, foliar-applied yeast extracts were found to reduce TSS in ‘Agiorgitiko’ cultivar [29], while another study on cv ‘Chambourcin’ reported that they led to a decrease in must pH [31]. These contrasting outcomes suggest that treatment dose and time may also influence the results. Indeed, in the study by Del Zozzo et al. [28], a proline-enriched yeast extract was applied 5 times to ‘Barbera’ cultivar until harvest. It was reported that TSS and maturity index decreased significantly (−2.09 Brix) in the yeast-treated vines, while pH, titratable acidity, tartaric acid, and malic acid contents remained unaffected. Our findings align with those reported in the literature. While different yeast concentrations induced minor changes in must composition, sugar, and organic acid content across both cultivars, no consistent pattern emerged.

### 2.3. Influences on Total and Individual Polyphenols

Table 3 presents the total and individual phenol results. In both cultivars, yeast treatments increased the total phenolic content, measured as total reducing capacity and expressed as gallic acid equivalents. In Early Cardinal, total phenol concentration ranged from 3300.93 mg kg^−1^ (Control) to 4206.45 mg kg^−1^ (Y3), and all yeast doses had positive effects at varying levels. Total phenol content increased in parallel with increasing yeast concentration but decreased slightly at the highest dose of 4 kg ha^−1^ (Y4). In this cultivar, individual phenols exhibited divergent accumulation patterns compared to total phenols. Epicatechin, chlorogenic acid, procyanidin-A1, and procyanidin-C1 did not differ significantly among yeast doses. Gallic acid and caftaric acid concentrations decreased significantly in all treatments relative to the control, while catechin, procyanidin-B1, and procyanidin-A2 also declined to varying degrees across all yeast applications. In contrast, epicatechin gallate increased significantly in Y2 and Y3. The flavonols rutin and quercetin-3-glucoside showed a similar trend, decreasing in the Y1 and Y2 treatments. Overall, we found no consistent trend in the responses of individual phenolic compounds in this cultivar, suggesting that the measured changes were not uniform across the phenolic compounds quantified in this study. Because we did not measure gene expression or enzyme activity, these results describe changes in the concentrations of the compounds analyzed and do not demonstrate activation of the phenylpropanoid pathway.

In Trakya Ilkeren, all the treatments significantly increased the total phenol content of the berries. Total phenol content was 2761.30 mg kg^−1^ in the control, while Y4 increased it to 3891.02 mg kg^−1^ and Y3 to 3845.36 mg kg^−1^. Considering the total phenol content, the best results were obtained at a dose of 4 kg ha^−1^ (Y4) in Trakya Ilkeren. Individual phenols, however, remained quite stable, as did anthocyanins. Catechin, chlorogenic acid, caftaric acid, rutin, quercetin-3-glucoside, procyanidin-C1, and procyanidin-A2 did not differ significantly between treatments. The Y1 dose yielded the highest levels of epicatechin, epicatechin gallate, procyanidin-A1, and procyanidin-B1. Although there were some differences in gallic acid levels, no difference was significant compared with the control. In this cultivar, although total phenols increased significantly, most individual phenols analyzed did not exhibit a corresponding change. These findings may be attributed to the possible accumulation of several flavonols, phenolic acids, and stilbenes (particularly resveratrol), which were not included in the present study and could have contributed to the overall increase in phenol concentration. Indeed, previous findings showed that yeast-derived elicitors differentially affected various phenolic subclasses.

Although many researchers reported that yeast-derived extracts stimulate the biosynthesis of secondary metabolites in grapevines, their effects may vary by concentration and cultivar. A 1 kg ha^−1^ yeast dose has been reported to increase total flavonoid content in ‘Merlot’ and phenolic maturity parameters, total phenol, and resveratrol content in ‘Syrah’ grapes and wines [24,32]. Gil-Muñoz et al. [33] emphasized that, although results varied by cultivar and vintage, yeast extracts effectively increased stilbene content in grapes and wines of ‘Monastrell’ and ‘Tempranillo’, grown in Spain. In another comprehensive study conducted in ‘Tempranillo’, it was reported that yeast treatment did not affect the total or individual flavonols (myricetin, quercetin, laricitrin, kaempferol, isorhamnetin, and syringetin), flavanols (catechin, epigallocatechin, and epicatechin-3-gallate), or phenolic acids (gallic acid, *trans-*caftaric acid, *trans-* and *cis-*coutaric acids, caffeic acid, *p-*coumaric acid) of grapes and wines, but increased the trans-resveratrol and total stilbene content of grapes [22].

### 2.4. Influences on Total and Individual Anthocyanins and Skin Color Parameters

Table 4 shows effects of yeast treatments on total anthocyanins and skin color, and Table 5 shows individual anthocyanins. In Early Cardinal, yeast treatments had a limited effect on total anthocyanin content, and variations among yeast doses were not statistically significant. The findings varied from 208.69 mg kg^−1^ (Y4) to 176.58 mg kg^−1^ (Y3). Color parameters were not affected by the treatments, except for minor changes together with the anthocyanin concentration. The most significant increase was observed at the 4 kg ha^−1^ dose (Y4) for anthocyanins (208.69 mg kg^−1^) and CIRG (Color Index for Red Grapes) (5.16); however, these increases were not statistically significant. The amounts of individual anthocyanins and their ratios are as important as total anthocyanins in determining the skin color of a given cultivar. Accordingly, in cv Early Cardinal, with claret red–violet skin color, the anthocyanin found in the highest amount was peonidin*-3-O-*glucoside, which gives a magenta color (104.44–74.02 mg kg^−1^), followed by malvidin*-3-O-*glucoside, which gives a purple–violet color (60.45–45.16 mg kg^−1^). Peonidin*-3-O-*glucoside constituted 50.6%, and malvidin*-3-O-*glucoside constituted 30.7% of the total anthocyanins in this cultivar (Figure 1). Cyanidin*-3-O-*glucoside (reddish-purple-magenta), petunidin*-3-O-*glucoside (blue–purple), and delphinidin*-3-O-*glucoside (dark blue) remained at much lower levels. Malvidin*-p-*coumaroyl-glucoside ranged from 12.40 to 9.35 mg kg^−1^, while malvidin-acetate-glucoside was detected at very low levels. *p-*coumaric forms of peonidin*-3-O-*glucoside and petunidin*-3-O-*glucoside were also very low. In Early Cardinal, all treatments, except the lowest dose (Y1), significantly increased malvidin*-3-O-*glucoside levels (Table 5). The increase in malvidin, which is present at the highest levels in blue–black grapes, was considered a promising result for the coloration problem that occurs in some years in Early Cardinal. The highest dose (Y4) significantly increased peonidin*-3-O-*glucoside, which is the most abundant form in this cultivar. Statistically significant increases in petunidin*-3-O-*glucoside were also observed at doses of Y2, Y3, and Y4. Overall, anthocyanins in the *3-O-*glucoside form reached their highest levels at the Y4 dose, although the difference was not statistically significant. No specific and consistent effect of the treatments on the *p-*coumaric forms of anthocyanins could be determined. While malvidin*-p-*coumaric-glucoside showed a slight decrease at Y3, other doses provided slight increases that were not statistically significant. The Y1 dose significantly increased the petunidin*-p-*coumaric-glucoside form, whereas peonidin*-p-*coumaric-glucoside and malvidin-acetate-glucoside did not differ significantly among treatments. The highest yeast dose increased malvidin and peonidin levels, the two most abundant anthocyanins in Early Cardinal, which was considered a positive result for increasing skin color intensity, although this was not reflected in CIRG.

Similarly, in Trakya Ilkeren, treatments had minimal effects on total anthocyanin content. The findings ranged from 223.75 mg kg^−1^ (Y3) to 236.33 mg kg^−1^ (Y1). Consistent with anthocyanin concentration, berry skin color generally showed no significant differences. Only Y2 and Y3 doses decreased b* and hue values. Although not statistically significant, the Y1 dose had a slightly positive effect on total anthocyanins (Table 4). In this cultivar, which has a blue-black skin color, the dominant anthocyanin was, as expected, malvidin*-3-O-*glucoside (131.22–122.57 mg kg^−1^), which is responsible for a purple-violet color and constituted 60.2% of the total anthocyanins in Trakya Ilkeren (Figure 1). Malvidin was followed by peonidin*-3-O-*glucoside (19.47–17.02 mg kg^−1^) and petunidin*-3-O-*glucoside (11.70–10.21 mg kg^−1^). Delphinidin*-3-O-*glucoside and cyanidin*-3-O-*glucoside were detected in low levels. Malvidin*-p-*coumaric-glucoside ranged from 36.78 to 32.12 mg kg^−1^ and constituted 16% of the total anthocyanins. Malvidin-acetate-glucoside, petunidin*-p-*coumaric-glucoside, and peonidin*-p-*coumaric-glucoside were detected in low levels (Table 5). The effects of yeast treatments on individual anthocyanins remained limited, consistent with the total anthocyanin concentrations. Although there were small increases and decreases across doses, these effects could not reach statistical significance. The same was true for the *p-*coumaric and acetate forms of anthocyanins. In this cultivar, yeast treatments positively affected other phenolic components rather than anthocyanins or skin color parameters.

In the present study, while yeast treatments caused some changes in total and individual anthocyanin content, the overall anthocyanin pattern remained stable in both cultivars, consistent with reports on ‘Sangiovese’, ‘Tempranillo’, and ‘Agiorgitiko’ [22,27,29]. However, some studies have yielded contradictory results. Different yeast doses treated by Liu et al. [34] led to different outcomes across two cultivars. The authors determined that yeast treatment increased total anthocyanin content by 18% and some individual anthocyanins (peonidin, petunidin, cyanidin, and delphinidin) by 15–52% in Cabernet Sauvignon. In contrast, in ‘Marselan’, total anthocyanins decreased by 33%, and individual anthocyanins decreased by 20–85%. The authors stated that the response variations may reflect cultivar-specific genetic differences. While Villangó et al. [32] and Giacosa et al. [35] reported that yeast treatment increased the total anthocyanins in ‘Nebbiolo’ and ‘Syrah’ grapes, Portu et al. [22] showed that yeast extracts had a limited effect on the anthocyanin concentration of grapes and wines of ‘Tempranillo’ and increased malvidin-*3-O-*glucoside and peonidin*-3-O-*glucoside-acetate in the berries, but did not affect the color intensity of wines. Kogkou et al. [29] observed that yeast treatment at a 1 kg ha^−1^ dose did not affect the total or individual anthocyanin content, or the chromatic properties of the wines. They also noted that in semi-arid climates such as Southern Greece, where the study was conducted, higher concentrations or more frequent yeast applications might be necessary to achieve the expected responses. A limited number of studies on table grape cultivars prone to insufficient coloration have yielded conflicting results. Crupi et al. [13] reported that yeast extracts (1 kg ha^−1^) increased total and individual anthocyanins in Red Globe, Scarlotta Seedless, and Crimson Seedless, and that this increase was maintained after three weeks of cold storage. Conversely, Petoumenou and Patris [26] reported that yeast treatment (1.5 kg L^−1^) did not significantly affect the total anthocyanin content of Crimson Seedless, even slightly reduced it, and decreased CIRG values. Although these studies used the same yeast extract, treatment time, method, and similar concentrations, their conflicting results suggest that the effect of yeast extracts may vary with ecology, vintage, and genotype. Our results regarding anthocyanin concentration and composition are similar to those reported by Kogkou et al. [29] and Petoumenou and Patris [26]. In our study, 4 yeast concentrations were tested, and the treatment effects on total and individual anthocyanins and color characteristics were lower than expected in both grape cultivars. It is known that anthocyanin biosynthesis can be inhibited above 30 °C [7,36,37]. Therefore, yeast treatments from the onset of véraison stimulate the biosynthetic pathway, thereby increasing anthocyanins in berries during periods when temperatures are usually lower and achieving a balance between technological and phenolic maturity [13]. However, in the region where our study was conducted, ripening occurs earlier because of temperatures that are quite high. It was thought that the effect of yeasts on anthocyanin accumulation might have been limited, particularly because of the relatively short period between véraison and maturity. Nevertheless, this is a tentative explanation: berry or canopy temperatures were not recorded in the present trial, and interpretations were based on monthly means from a meteorological station. The role of high temperature in limiting anthocyanin accumulation therefore remains a hypothesis that requires more detailed analysis.

### 2.5. Influences on Antioxidant Activity

Table 6 presents the effects of yeast treatments on antioxidant activity. In Early Cardinal, DPPH (1,1-diphenyl-2-picrylhydrazyl) and FRAP (ferric reducing antioxidant power), which indicate antioxidant activity, reached their highest levels at the 4 kg ha^−1^ dose (Y4), paralleling the trend in anthocyanin content. The 2 kg ha^−1^ dose (Y3) also led to small increases. Notably, the lowest values were observed at the 0.5 kg ha^−1^ dose (Y1). In Trakya Ilkeren, all yeast concentrations, except the Y2 treatment, increased antioxidant activity at varying levels. The increases achieved at Y1 for DPPH and at Y1 and Y3 doses for FRAP were statistically significant. At the Y2 treatment, antioxidant activity decreased compared to the control samples.

Some studies have indicated that foliar yeast treatments did not significantly affect the antioxidant activity of wine grapes and wines [22,29]. Conversely, Tahmaz et al. [38] determined that yeast extracts in cv ‘Nero D’Avola’ increased antioxidant capacity (DPPH and ascorbate peroxidase) and antioxidant enzyme activity (catalase, superoxide dismutase, and ascorbate peroxidase) under salt stress, strengthening the defense mechanism of grapevines. Šuklje et al. [39] also reported that yeast extract on ‘Sauvignon Blanc’ increased GSH (glutathione) content, an important antioxidant present in grapes, musts, and wines. Our findings are consistent with previous studies reporting that yeasts increase antioxidant activity. Although the grape cultivars in our study exhibited different patterns of antioxidant activity, we observed a positive effect of yeast extract in both cultivars.

### 2.6. Multivariate Analysis

The multivariate analyses presented in this section are exploratory and descriptive. Each cultivar had approximately 51 variables and only 15 observations (five treatments × three replicates), and the 95% confidence ellipses are derived from three replicates per treatment. Therefore, ordination, the heatmap, and the correlation coefficients are used only to summarize the structure of the dataset and generate hypotheses; they do not constitute independent statistical confirmation of treatment effects or of mechanistic relationships. The multivariate structure of the dataset was descriptively consistent with the univariate findings reported above, in that the two cultivars showed different patterns of response, a tendency also reported by Portu et al. [22], Kogkou et al. [29], and Liu et al. [34]. In ‘Early Cardinal’, the first two principal components accounted for 45.9% of the total variance and produced a separation among treatments (Figure 2a). The control clustered near the origin, whereas the yeast doses diverged along distinct directions: Y4 was displaced toward the positive PC1–PC2 quadrant, defined by pH, fructose, maturity index (MI), and the major anthocyanins peonidin-glucoside and malvidin*-3-O-*glucoside*-p-*coumaric, while Y1 loaded strongly on the phenolic acids (gallic and caftaric acid), procyanidin-B1, and petunidin*-3-O-*glucoside*-p-*coumaric. Y3 separated in the opposite direction, associated with organic acids (tartaric, citric, ascorbic) and the antioxidant descriptors DPPH and FRAP. This ordination visually reinforces the descriptive pattern already seen in the univariate data, in which the treatments were associated with different subsets of variables rather than with a uniform shift in all parameters; given the low observation-to-variable ratio, this ordination should be regarded as a summary of the dataset rather than as evidence of a specific regulatory mechanism. A comparable variability among phenolic subclasses was reported by Portu et al. [22] and the dose-dependent responses observed by Del Zozzo et al. [28]. In ‘Trakya Ilkeren’, the first two components explained a lower proportion of the variance (35.9%), and the treatment ellipses overlapped more extensively (Figure 2b), indicating a more conservative response in this already dark-skin-colored cultivar. The only clearly resolved group was Y2, which separated along negative PC1 in association with flavan-3-ols and condensed tannins (catechin, epicatechin, procyanidins A2 and B1), whereas Y1 and Y4 were grouped towards the antioxidant and color axis (FRAP, DPPH, a*, b*). This pattern mirrors the univariate observation that, in ‘Trakya Ilkeren’, the benefit of yeast treatment was expressed through phenolic and antioxidant components rather than through anthocyanin or skin color and is congruent with reports that yeast elicitors act on secondary metabolism without displacing the anthocyanin balance in dark-colored genotypes [27,29].

The heatmap (Figure 3) summarized these dose–parameter relationships across all 51 variables and highlighted the absence of a single dominant dose. In ‘Early Cardinal’, Y4 emerged as the most influential treatment, coinciding with above-average values for maturity index (MI), total monomeric anthocyanins (TMA), several *3-O-*glucosides (peonidin-, delphinidin-, cyanidin-), and both antioxidant assays, whereas Y1 was characterized by elevated flavan-3-ols and phenolic acids. In ‘Trakya Ilkeren’, the strongest positive signals were distributed among Y1 and Y2 for individual phenols (epicatechin, epicatechin gallate, procyanidins) rather than for anthocyanins, visually confirming the decoupling of the phenolic and anthocyanic responses in this cultivar. This genotype-driven redistribution of secondary metabolites, without a consistent single-dose effect, aligns with the variable outcomes across different ecologies and vintages reported by Villangó et al. [32], Giacosa et al. [35], and Gil-Muñoz et al. [33].

The correlation matrices (Figure 4) show several associations that appeared in both cultivars. As these coefficients were calculated on a small dataset (*n* = 15 per cultivar) that combined treatments and replicates, they describe linear associations only within the present dataset and should not be interpreted as causal or mechanistic relationships. As expected, MI was strongly and negatively correlated with titratable acidity (r = −0.94 in ‘Early Cardinal’; r = −0.88 in ‘Trakya Ilkeren’), and the two antioxidant assays were tightly coupled (DPPH and FRAP, r = 0.91 and r = 0.78, respectively), confirming the consistency of the measurements. In ‘Early Cardinal’, antioxidant activity was positively linked to anthocyanin accumulation and ripening: peonidin*-3-O-*glucoside, the dominant anthocyanin of this cultivar, correlated positively with MI (r = 0.78), TMA (r = 0.68) and TSS (r = 0.61), while color intensity (CIRG) tracked yield and the a* coordinate (r = 0.58 and r = 0.61). In ‘Trakya Ilkeren’, by contrast, DPPH and FRAP were negatively correlated with TSS (r = −0.65 and r = −0.70), indicating that increases in the antioxidants obtained with yeast treatment were largely independent of sugar accumulation; here, TMA was governed by anthocyanins malvidin*-3-O-*glucoside and peonidin*-3-O-*glucoside (r = 0.74 for both), and malvidin*-3-O-*glucoside was further associated with berry weight (r = 0.69). Taken together, the multivariate evidence suggests that, within the present dataset, the yeast treatments were not associated with a uniform change in a single quality trait but rather with different combinations of sugars, acids, anthocyanins, and phenolics in each cultivar. Confirmation of this pattern would require a dataset with a larger sample size relative to the number of variables and across additional sites and seasons [13,34].

## 3. Materials and Methods

### 3.1. Plant Material and Experimental Location

This study was conducted during the 2025 growing season on ‘Early Cardinal’ (mutant of ‘Cardinal’ [Flame Tokay × Alphonse Lavallée]) and ‘Trakya Ilkeren’ (Alphonse Lavallée × Perlette) table grapes (*Vitis vinifera* L.). The trial vines are grown in the Experimental Vineyard of the Department of Horticulture at Çukurova University in Adana, Türkiye (37°01′48″ N, 35°22′49″ E, 70 m a.s.l.). Early Cardinal has red–violet skin and is typically prone to poor coloration. Trakya Ilkeren has blue–black skin. Both cultivars are early-ripening and used for fresh consumption. The plots were planted in 2017 with 2 × 3.5 m row spacing. Grapevines were grafted onto 1103P rootstock and trained to a bilateral cordon. The vineyard soil was clay–loam, alkaline, and calcareous. The research region has a typical Mediterranean climate, with warm, humid conditions and limited precipitation. The main climatic conditions of the experimental area from dormancy to harvest time in 2025 were obtained from the Turkish State Meteorological Service (Table 7).

### 3.2. Trial Design and Treatments

The experiment was conducted according to a randomized block design with three replicates, each consisting of four vines. The trial was carried out in a single experimental vineyard during a single growing season (2025); therefore, the results reflect the site- and vintage-specific conditions of this experiment. The vines were spur-pruned from 2–3 buds during the dormant period. Lalvigne^®^ MATURE (Lallemand Inc., Montreal, QC, Canada) was used as yeast extract. Lalvigne^®^ MATURE is a formulation of 100% natural, inactivated wine yeast (*Saccharomyces cerevisiae*) derivatives, designed to improve phenolic maturity and overall grape quality. The water-suspended yeast formulation was applied to the whole leaf surface with a backpack sprayer. Two treatments were performed, the first at the beginning of véraison and the second 10 days later. The treatments were conducted on 4 and 14 June at Early Cardinal and on 6 and 16 June at Trakya Ilkeren. We determined the doses according to the company’s instructions and previous studies [13,27,29,35]. However, we also used doses lower and higher than those previously reported to assess their effectiveness. The doses were determined as follows: 0.5 kg ha^−1^ (Y1), 1 kg ha^−1^ (Y2), 2 kg ha^−1^ (Y3), and 4 kg ha^−1^ (Y4). The control vines (C) were sprayed only with a water suspension of a surfactant. Standard cultural practices (leaf removal, shoot tipping, and removal of water sprouts and suckers) for table grapes were performed according to local practices. Appropriate fungicides were used to control powdery mildew and downy mildew. Vines were managed with no irrigation. Grapes were manually harvested at commercial maturity, approximately 15–17 °Brix [40] (Early Cardinal on 21 June, Trakya Ilkeren on 23 June). After harvest, samples for phytochemical analysis were immediately frozen and stored at −20 °C until analysis. The following measurements and analyses were conducted to assess the effects of the treatments.

### 3.3. Cluster and Berry Characteristics

Five clusters were used in each replicate to determine cluster weight (g), cluster width, and length (cm). Berry weight (g), berry width, and length (mm) were determined on berries randomly selected from five clusters in each replicate. Berry weight was calculated as the average of 100 berries; berry width and length were measured using a digital caliper on 50 berries per replicate. Berry skin color was measured at the equatorial position of the berries according to the CIELab color system.

Similarly, we used five clusters for color measurements per replicate. L*, a*, and b* were determined using a Konica Minolta CR200 (Tokyo, Japan). Then, hue angle and chroma were calculated and converted to the CIRG (Color Index for Red Grapes) index according to Carreño et al. [41]. Since the hue values of both cultivars were between 360° and 270°, to facilitate the calculations, hue angle was considered negative (e.g., 344° was taken as −16°) [42].

### 3.4. Yield and Ripeness Parameters

Yield per vine (kg) was calculated in total for 12 vines (4 vines per replicate) by multiplying the total number of clusters per vine by the average cluster weight. Total soluble solids (TSS, °Brix), titratable acidity (g 100 mL^−1^), and pH were determined in must from 100 randomly selected berries from five clusters in each replicate. TSS was determined using a digital refractometer (H196801, Hanna Instruments, Woonsocket, RI, USA). Titratable acidity (TA) was determined by titrating the must with 0.1 N NaOH to a pH of 8.1 (pH330i-WTW, Weilheim, Germany) and expressed as tartaric acid equivalents. The maturity index (MI) was calculated by dividing the TSS by titratable acidity.

### 3.5. Phytochemical Analyses

For phytochemical analyses, a total of 5 clusters were sampled from 4 vines in each replicate. A total of 300 g of berry samples were randomly taken from the clusters, placed in plastic zip-lock bags, immediately frozen, and stored at −20 °C until analysis.

#### 3.5.1. Extraction

The grape samples stored at −20 °C were thawed overnight in a refrigerator (+4 °C). The berry samples were then homogenized in a high-speed shredder (Waring, Commercial, Torrington, CT, USA). Five g of homogenate was transferred to a 50 mL centrifuge tube, and 45 mL of an 80% (*v*/*v*) MeOH solution was added. The centrifuge tube was then vortex-mixed for 30 s and centrifuged (6000 rpm, 4 °C, 10 min). The resulting stock extract was utilized for total phenol, total anthocyanin, and antioxidant activity analyses.

#### 3.5.2. Determination of Total Phenolic Content (TPC)

Total phenolic content was determined with some modifications of the Folin–Ciocalteu spectrophotometric method previously described by Abdullakasim et al. [43]. For the analysis, 100 μL of the stock extract was transferred to a test tube, 200 μL of Folin–Ciocalteu reagent and 3000 μL of distilled water were added, and the mixture was left to stand for 10 min. After the designated period, 100 μL of Na_2_CO_3_ (20%, *w*/*v*) was added, and the mixture was kept in the dark at room temperature (~22 °C) for 2 h. Subsequently, the absorbance of the solution was measured at 765 nm using a spectrophotometer (PerkinElmer Lambda 25 UV/VIS, PerkinElmer, Inc., Shelton, CT, USA) against the blank. The quantity of phenolic compounds, corresponding to the measured absorbance value, was determined using the standard curve equation prepared with gallic acid. The total phenolic content of the samples was expressed as mg GAE kg^−1^ fresh weight (FW).

#### 3.5.3. Determination of Total Monomeric Anthocyanin Content (TMA)

Total monomeric anthocyanin content was determined using the pH differential method described by Giusti and Wrolstad [44]. For total monomeric anthocyanin analysis, 0.5 mL of the stock extract was added to two glass tubes; 2.5 mL of pH 1.0 potassium chloride solution (0.025 M) was added to one tube and 2.5 mL of pH 4.5 sodium acetate solution (0.4 M) to the other, and then the mixtures were shaken. The dilutions were then allowed to equilibrate for 15 min at room temperature (~22 °C). The absorbance of the equilibrated samples was measured against a blank cell containing distilled water using 1 cm path-length disposable cuvettes at 520 nm (λ_vis_-max) for anthocyanin content and 700 nm for haze correction. The absorbance (A) of the diluted sample was then calculated as follows:

$$A=\left(A_{520\mathrm{nm}}-A_{700\mathrm{nm}}\right)pH1-\left(A_{520\mathrm{nm}}-A_{700\mathrm{nm}}\right)pH4.5$$

The total monomeric anthocyanin (TMA) content in the sample was calculated according to the following formula:

$$TMA\left({\mathrm{mg}\;\mathrm{kg}}^{-1}FW\right)=\frac{\left(A\times MW\times DF\right)\;\times 1000}{\varepsilon \times l}$$

in which malvidin*-3-O-*glucoside molecular weight (MW = 493.5 g mol^−1^), the dilution factor (DF), the molar absorptivity constant (ε = 29,600 L cm^−1^ mol^−1^), and l = path length (1 cm) were used. Results were expressed as mg of malvidin-3-glucoside equivalents per kg fresh weight. All spectrophotometric analyses were performed using a UV-visible spectrophotometer (Perkin Elmer Lambda 25 UV/VIS, PerkinElmer, Inc., Shelton, CT, USA).

#### 3.5.4. Determination of Antioxidant Activity

For antioxidant activity, the DPPH* (1,1-diphenyl-2-picrylhydrazyl) free radical-scavenging method proposed by Klimczak et al. [45] and the FRAP (ferric reducing antioxidant power) method proposed by Benzie and Strain [46] were used, with some modifications. For DPPH* analysis, 100 μL of the stock extract was added to 3000 μL of DPPH* radical (0.025 g L^−1^ in 80% methanol) and mixed by the vortex. The samples were incubated in the dark for 60 min to allow the reaction to reach equilibrium. After this period, the absorbance of the samples was measured at 515 nm using the spectrophotometer (Perkin Elmer Lambda 25 UV/VIS, PerkinElmer, Inc., Shelton, CT, USA). In the FRAP method, 2900 μL of FRAP reagent was added to 100 μL of stock extract, and the mixture was left in the dark for 1 h to equilibrate. Subsequently, the absorbance of the samples was measured at 593 nm on a spectrophotometer. Trolox equivalents of antioxidant activity values obtained by DPPH* and FRAP methods were also determined. Calibration curves were obtained by using Trolox solutions at different concentrations. The antioxidant activity of the samples was calculated from the equations derived from the curves and expressed as mg Trolox equivalents per kg fresh weight (mg TE kg^−1^ FW).

#### 3.5.5. Individual Phenolic Compounds and Anthocyanins

Phenolic compounds were analyzed by HPLC (high-performance liquid chromatography) using the method adapted from Agcam [47]. The stock extract was filtered with 0.45 µm membrane filters (PTFE), and then 20 µL of the sample was injected into the HPLC-PDA (Shimadzu, Kyoto, Japan) equipped with a C18 XTERRA RP (5 µm, 4.6 × 250 mm, Waters, Milford, MA, USA) column. The chromatographic conditions, mobile-phase gradient elution, and method validation parameters were described by Agcam [47]. The separated peaks were identified and quantified using the external standard method. Identification of phenolic compounds was accomplished by comparing retention times, elution sequences, and UV-Vis absorption spectra (200–600 nm) with those of authentic commercial standards. Quantitative chromatograms were monitored at targeted analytical wavelengths: 280 nm (flavan-3-ols/hydroxybenzoic acids), 320 nm (hydroxycinnamic acids), 355 nm (flavonols), and 520 nm (anthocyanins). Standard calibration curves were established across a concentration range of 0.1–50 mg L^−1^. Method validation metrics demonstrated high linearity and sensitivity across all analyzed classes: phenolic acids (coefficients of determination-(*R*^2^) = 0.9993–0.9998; limit of detection-(LOD) = 0.010–0.077 mg L^−1^; limit of quantification-(LOQ) = 0.012–0.097 mg L^−1^), anthocyanins (*R*^2^ = 0.9985–0.9997; LOD = 0.060–0.104 mg/L; LOQ = 0.082–0.119 mg L^−1^), and non-anthocyanin flavonoids (*R*^2^ = 0.9989–0.9996; LOD = 0.053–0.086 mg L^−1^; LOQ = 0.072–0.104 mg L^−1^). The results were expressed as mg kg^−1^ FW. The representative chromatograms of the phenolic compounds are shown in the Supplementary File (Figure S1A–D).

#### 3.5.6. Organic Acids and Sugars

Organic acid and sugar contents of grape samples were evaluated using the method of Sturm et al. [48]. Berry samples were homogenized in a high-speed shredder (Waring, Boerne, TX, USA). After that, 1 g of homogenate was transferred to a 15 mL centrifuge tube and made up to 10 g with deionized water. Samples were centrifuged at 4000 rpm and 4 °C for 10 min. Supernatant was filtered (0.45 μm PVDF filter) and injected into the HPLC system (Shimadzu, Kyoto, Japan). The mobile phase, injection volume, flow rate, wavelength, column, temperature, and detector for the HPLC-PDA/RID conditions were 5 mM H_2_SO_4_ (isocratic flow), 20 μL, 0.3 mL/min, 210 nm–244 nm, Concise-Coregel/Ion 300, 30 °C, and Photo Diode Array (PDA)-Refractive Index (RID) (Shimadzu Corp., Kyoto, Japan), respectively. Certified commercial standards were used to detect and quantify organic acid and sugar peaks. Organic acids (citric, malic, tartaric, and ascorbic acids) and simple sugars (fructose, glucose, and sucrose) were identified by comparing their retention times, elution sequences, and spectral characteristics with those of authentic commercial standards. Organic acids were monitored via PDA detection at 210 nm (citric, malic, and tartaric acids) and 244 nm (ascorbic acid). Quantification was performed using external calibration curves constructed across concentration ranges of 1–100 mg L^−1^ for organic acids and 0.5–5 g/100 mL for individual sugars. Method validation metrics confirmed high linearity and sensitivity across all analytes. For organic acids, LOD and LOQ ranged from 0.085 to 0.116 mg/L and 0.142 to 0.190 mg/L, respectively, with *R*^2^ spanning 0.9991–0.9995. For monosaccharides (glucose and fructose), LOD and LOQ values ranged from 0.012 to 0.015 g 100 mL^−1^ and 0.021 to 0.073 g 100 mL^−1^, respectively, with *R*^2^ values ranging from 0.9994 to 0.9999. Due to the lower concentration of sucrose in the sample matrix, sucrose quantification in real samples was performed using a dedicated low-range calibration curve (0.005–0.5 g 100 mL^−1^), yielding an LOD of 0.00027 g 100 mL^−1^ and an LOQ of 0.00034 g 100 mL^−1^ (*R*^2^ = 0.9998). The results were expressed as g 100 g^−1^ FW (sugar compounds) and mg kg^−1^ FW (organic acids). The representative chromatograms of the identified organic acids and sugar compounds are provided in the Supplementary File (Figures S2 and S3A,B).

### 3.6. Statistical Analysis

All analyses were performed using three independent biological replicates (*n* = 3). Each biological replicate was used independently throughout all experimental procedures and physical and biochemical analyses. Results are expressed as mean ± standard deviation. The normality assumption for performing ANOVA was evaluated by the Kolmogorov–Smirnov normality test. After the normality assumption provided, the data of each cultivar were analyzed with a linear model appropriate for the randomized complete block design, in which treatment (C, Y1, Y2, Y3, Y4) was included as a fixed effect and block as a random effect, according to the model Yij = μ + τi + βj + εij, where μ is the overall mean, τi the fixed effect of the i-th treatment, βj the random effect of the j-th block, and εij the residual error. Analyses were performed in JMP (v13.00, SAS Institute Inc., Cary, NC, USA) software. The two cultivars were located in separate adjacent plots, treated on different dates and harvested on different dates; therefore, each cultivar was analyzed separately, and the cultivar × treatment interaction was not tested. Accordingly, differences observed between the two cultivars are described only qualitatively and are not presented as statistically tested cultivar-dependent responses. Differences between the treatments were compared using Fisher’s protected LSD (least significant difference) test at the 5% level. In accordance with the protected LSD procedure, mean separation letters are reported only for those parameters where global ANOVA F-test was significant (*p* ≤ 0.05); for parameters with *p* > 0.05, the LSD value is reported as NS and no letters are shown. To further explore the multiple relationships among treatments and parameters, principal component analysis (PCA), a correlation-based heatmap, and Pearson correlation analysis were additionally carried out for each cultivar in Python 3.12 using the scikit-learn (v1.5.0), pandas (v2.2.0), NumPy (v1.26.0), and Matplotlib (v3.9.0) libraries; prior to PCA, all variables were standardized (z-score, zero mean, and unit variance) and the treatment structure was visualized as biplots with 95% confidence ellipses, with treatment means expressed as z-scores computed across the five treatments within each cultivar for the heatmap, and linear associations among the main quality traits quantified by Pearson correlation coefficients (r) calculated on the full replicate dataset (*n* = 15 per cultivar), with |r| ≥ 0.51 corresponding to *p* < 0.05.

## 4. Conclusions

This study concluded that the two table grape cultivars examined here responded differently to foliar yeast treatment. Because each cultivar was analyzed separately and no cultivar × treatment interaction was tested, the difference is presented as a descriptive observation rather than as a statistically demonstrated genotype effect. Although yeast treatments did not create a statistically significant difference in vine yield, both cultivars recorded the highest yield with the highest yeast concentration (Y4). In Early Cardinal, all yeast doses improved cluster and berry characteristics, with Y1 treatment standing out for cluster weight and berry size. In Trakya Ilkeren, cluster weight increased with increasing yeast dose; however, it decreased again at the Y4 dose, and the Y3 treatment yielded the best results. The must composition remained generally stable across both varieties. Yeast treatments induced some changes in the sugars and organic acids of the must in both cultivars; however, no clear trend was observed.

The primary objective of yeast treatment in this study was to improve anthocyanin concentration and skin color; however, the effects were lower than expected. Although total anthocyanin content and berry skin color varied slightly, these effects did not reach statistical significance. In Early Cardinal, doses Y2, Y3, and Y4 significantly increased malvidin*-3-O-*glucoside and petunidin*-3-O-*glucoside, while Y4 treatment significantly increased peonidin*-3-O-*glucoside. In Trakya Ilkeren, anthocyanin concentration and composition remained stable, and the berries maintained their characteristic skin color in all the yeast doses. Anthocyanin biosynthesis can be inhibited at temperatures above 30 °C. Therefore, yeast treatments from the onset of véraison aim to stimulate the biosynthesis pathway and increase anthocyanin accumulation in berries. However, the relatively short period between véraison and maturity in our region may have limited the activity period of yeasts on anthocyanin biosynthesis and accumulation. Therefore, we recommend investigating earlier yeast applications, starting at berry set, and higher application frequency (three or more applications) in future experiments for early-ripening table grape cultivars. This might prolong the yeast activity on berry and leaf surfaces until véraison. However, this timing was not tested in the present trial and is proposed as a direction for future research based on the data from this study. Although no consistent increase in individual phenols was observed, all treatments increased the total phenol content compared to the control. In Early Cardinal, the Y3 treatment, and in Trakya Ilkeren, the Y3 and Y4 doses, produced the greatest increases in total phenols. According to DPPH and FRAP analyses, the highest antioxidant activity was observed in Early Cardinal with Y3 and Y4 doses, and in Trakya İlkeren with Y1 and Y3 treatments. These findings demonstrated that yeast treatment was associated with higher total reducing capacity and antioxidant activity under the warm conditions of this experiment. Because no molecular or enzymatic markers were measured, no conclusion could be drawn here about whether stress defense mechanisms were activated. However, the measured increases in secondary metabolites and the antioxidant activity were considered favorable outcomes for the phytochemical profile of table grapes. To the best of our knowledge, this is the first study to test four different doses of foliar yeast extracts on the early-ripening table grape cultivars ‘Early Cardinal’ and ‘Trakya Ilkeren’. Although no single yeast dose stood out among the four concentrations tested, the higher concentrations, namely 2 kg ha^−1^ (Y3) and 4 kg ha^−1^ (Y4), were generally more effective. In ‘Early Cardinal’, Y4 (4 kg ha^−1^) stood out as the most influential dose in terms of technological maturity, increasing total and major individual anthocyanins (peonidin*-3-O-*glucoside and malvidin*-3-O-*glucoside) and yielding the highest antioxidant activity (DPPH and FRAP). Meanwhile, the Y3 (2 kg ha^−1^) dose was the most effective for total phenol accumulation; Y1 (0.5 kg ha^−1^) and Y2 (1 kg ha^−1^) were more effective for cluster and berry traits. In ‘Trakya Ilkeren’, Y3 (2 kg ha^−1^) and Y4 (4 kg ha^−1^) were the most effective treatments for yield, cluster, and berry traits, total phenolics, and antioxidant activity. The present study has several limitations that can be optimized in future experimental designs. The experiment was conducted in a single vineyard during a single growing season; the results need to be confirmed across different ecological conditions and vintages before making any general recommendations. Repeating the experiment over two or three consecutive growing seasons across different sites or *terroirs* would help decouple vintage effects and climatic influences from genotype-dependent responses. Furthermore, including application timing (berry set and véraison) and frequency as separate experimental factors alongside dose in a factorial design would allow for evaluating potential timing × dose interactions. Besides these, monitoring leaf and berry temperatures may help assess the potential physiological impacts of yeast extracts on canopy microclimate in grapevines grown under high temperatures. Finally, complementary assessments, including gene expression of anthocyanin and flavonoid biosynthesis pathways and postharvest measurements during cold storage, would clarify whether the observed responses stem from metabolic regulation and remain stable until consumption.

Given the limited number of studies on table grapes, this study provides preliminary insights that may guide future research on improving the physical and chemical quality of table grapes under warm conditions by natural, sustainable methods with yeasts naturally present in grapevines.

## Figures and Tables

**Figure 1:**
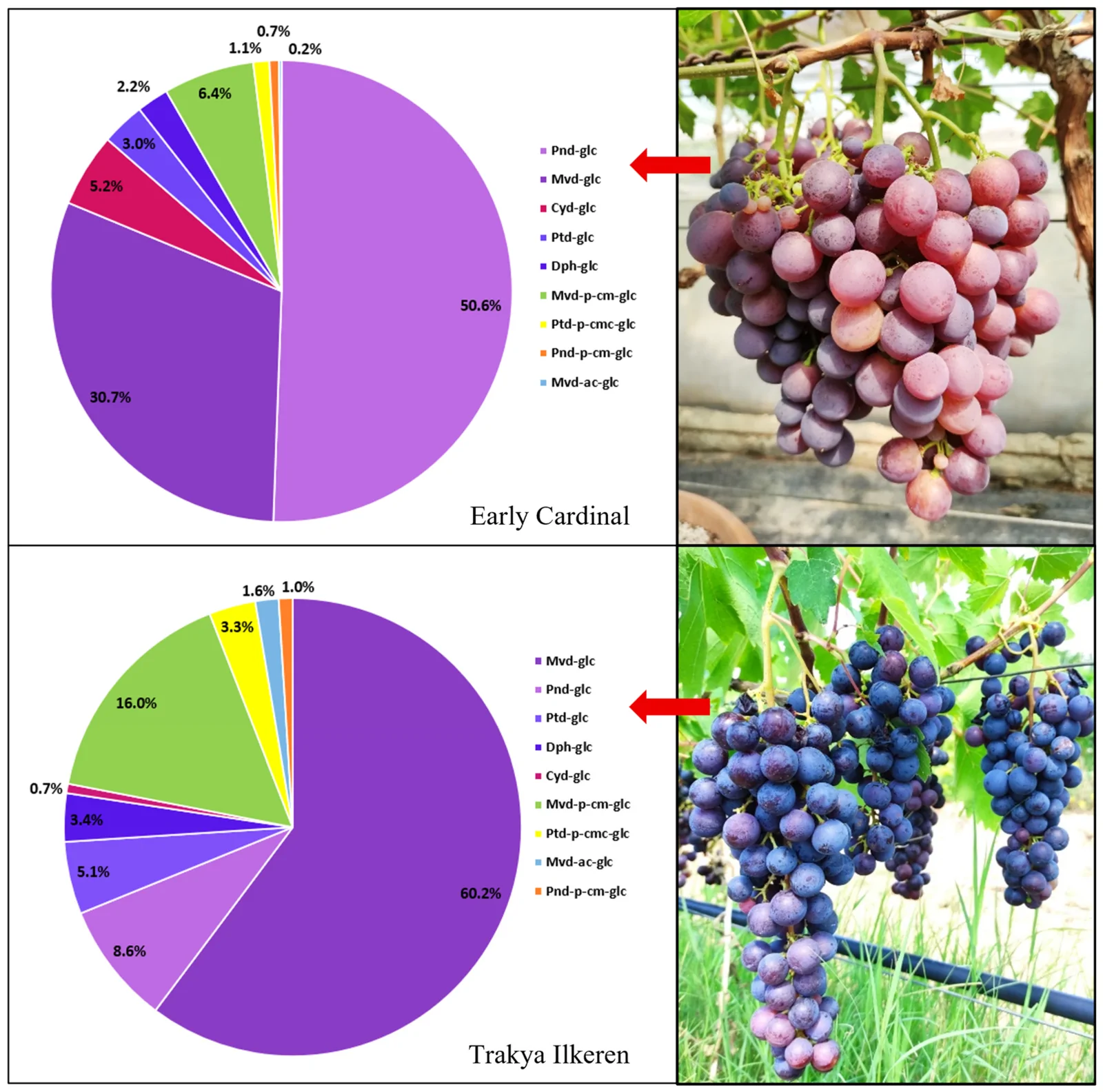
Clusters and anthocyanin profiles of ‘Early Cardinal’ and ‘Trakya Ilkeren’ grape cultivars. Mvd-glc: Malvidin*-3-O-*glucoside, Pnd-glc: Peonidin*-3-O-*glucoside, Ptd-glc: Petunidin*-3-O-*glucoside, Dph-glc: Delphinidin*-3-O-*glucoside, Cyd-glc: Cyanidin*-3-O-*glucoside, Mvd*-p-*cm-glc: Malvidin-3-O-glucoside*-p-*coumaric, Ptd*-p-*cm-glc: Petunidin*-3-O-*glucoside*-p-*coumaric, Pnd*-p-*cm-glc: Peonidin-3-O-glucoside*-p-*coumaric, Mvd-ac-glc: Malvidin*-3-O-*glucoside-acetate.

**Figure 2:**
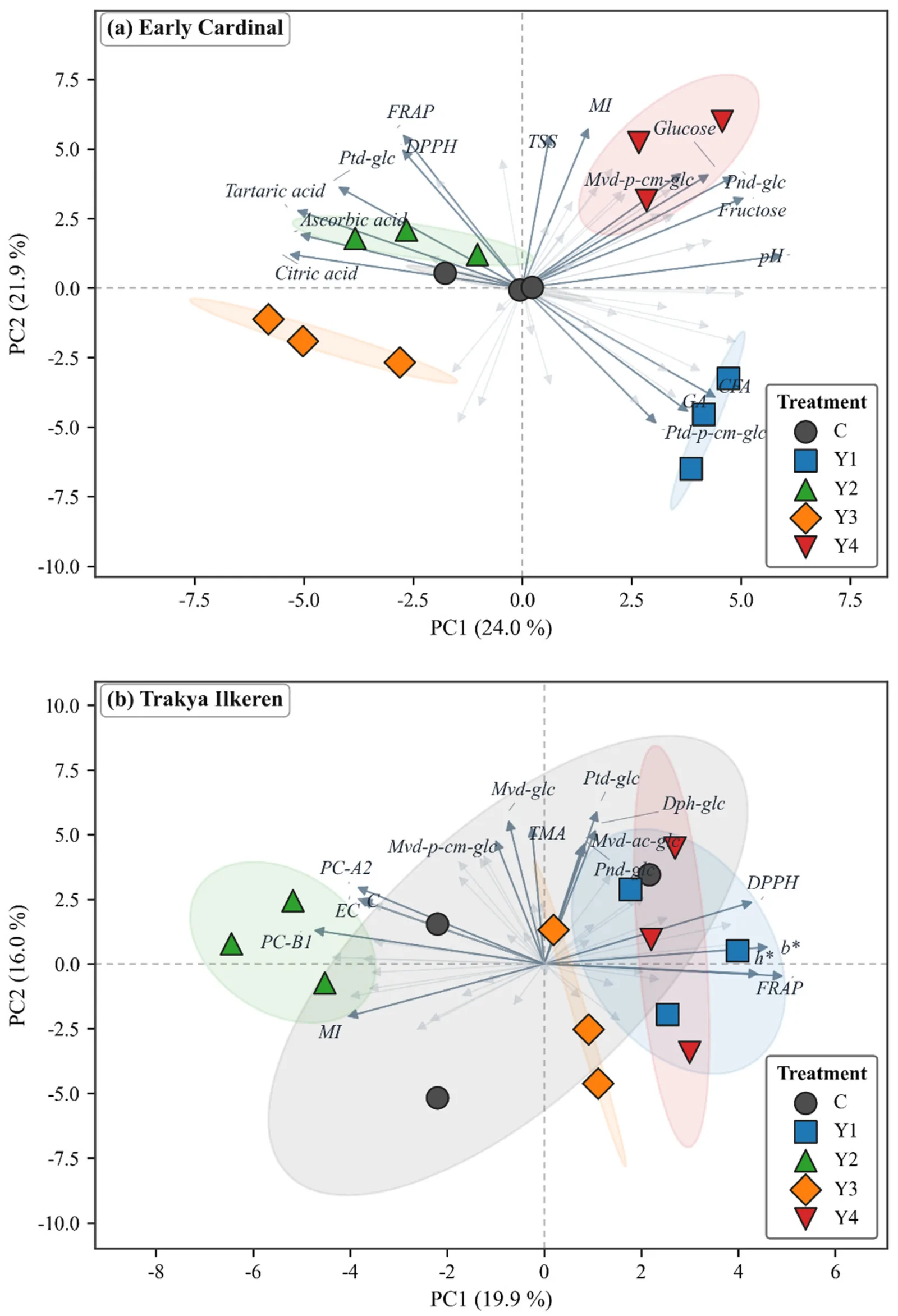
Principal component analysis (PCA) biplots of all measured yield, color, phenol, anthocyanin, and antioxidant parameters in (**a**) ‘Early Cardinal’ and (**b**) ‘Trakya Ilkeren’ berries treated with four foliar yeast doses (Y1: 0.5 kg ha^−1^, Y2: 1 kg ha^−1^, Y3: 2 kg ha^−1^, Y4: 4 kg ha^−1^) and an untreated control (C) (black circle). Symbols represent individual replicates (*n* = 3), shaded areas denote the 95% confidence ellipse of each treatment, which are based on only three replicates per treatment and are therefore indicative rather than inferential, and vectors indicate variable loadings. Variables were standardized (z-scored) prior to analysis; the first two principal components jointly explained 45.9% (**a**) and 35.9% (**b**) of the total variance. Mvd-glc: Malvidin*-3-O-*glucoside, Pnd-glc: Peonidin*-3-O-*glucoside, Ptd-glc: Petunidin*-3-O-*glucoside, Dph-glc: Delphinidin*-3-O-*glucoside, Mvd*-p-*cm-glc: Malvidin-3*-O-*glucoside-p-coumaric, Ptd*-p-*cm-glc: Petunidin*-3-O-*glucoside*-p-*coumaric, Mvd-ac-glc: Malvidin*-3-O-*glucoside-acetate, GA: gallic acid, CFA: caftaric acid, C: catechin, EC: epicatechin, PC-A2: procyanidin-A2, PC-B1: procyanidin-B1, TMA: total monomeric anthocyanins, DPPH: 1,1-diphenyl-2-picrylhydrazyl, FRAP: ferric reducing antioxidant power, TSS: Total soluble solids, MI: Maturity index.

**Figure 3:**
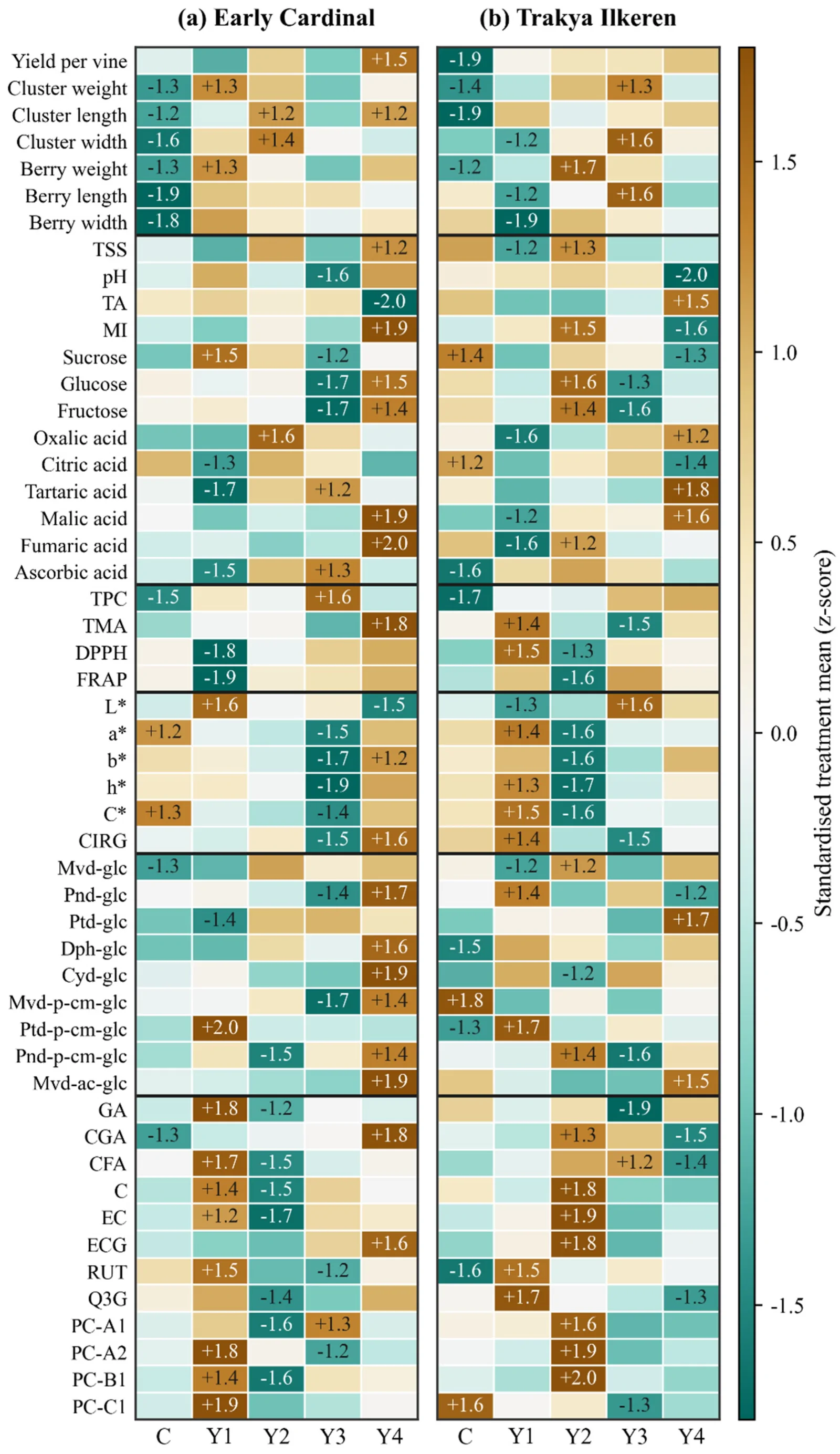
Heatmap of standardized treatment means for the 51 measured parameters in (**a**) ‘Early Cardinal’ and (**b**) ‘Trakya Ilkeren’. Colors range from teal (below the cultivar mean) to brown (above the cultivar mean); values shown in the strongly deviating cells (|z| ≥ 1.15) are the corresponding z-scores. Each cell represents the mean of three replicates expressed as a z-score computed across the five treatments within each cultivar. On the horizontal axis, yeast doses are represented as Y1: 0.5 kg ha^−1^, Y2: 1 kg ha^−1^, Y3: 2 kg ha^−1^, Y4: 4 kg ha^−1^, alongside an untreated control (C). The vertical axis represents the following parameters: TSS: Total soluble solids, TA: Titratable acidity, MI: Maturity index, TPC: total phenol content, TMA: total monomeric anthocyanins, DPPH: 1,1-diphenyl-2-picrylhydrazyl, FRAP: ferric reducing antioxidant power, L*: lightness, a*: red/green scale b*: yellow/blue scale, h*: hue, C*: chroma, CIRG: Color Index for Red Grapes, Mvd-glc: Malvidin*-3-O-*glucoside, Pnd-glc: Peonidin*-3-O-*glucoside, Ptd-glc: Petunidin*-3-O-*glucoside, Dph-glc: Delphinidin-3-O-glucoside, Cyd-glc: Cyanidin*-3-O-*glucoside, Mvd-p-cm-glc: Malvidin*-3-O-*glucoside*-p-*coumaric, Ptd*-p-*cm-glc: Pe-tunidin*-3-O-*glucoside*-p-*coumaric, Pnd*-p-*cm-glc: Peonidin*-3-O*-glucoside*-p-*coumaric, Mvd-ac-glc: Malvidin*-3-O-*glucoside-acetate, GA: gallic acid, CGA: chlorogenic acid, CFA: caftaric acid, C: catechin, EC: epicatechin, ECG: epicatechin gallate, RUT: rutin, Q3G: quercetin-3-glucoside, PC-A1: procyanidin-A1, PC-A2: procyanidin-A2, PC-B1: procyanidin-B1, PC-C1: procyanidin-C1.

**Figure 4:**
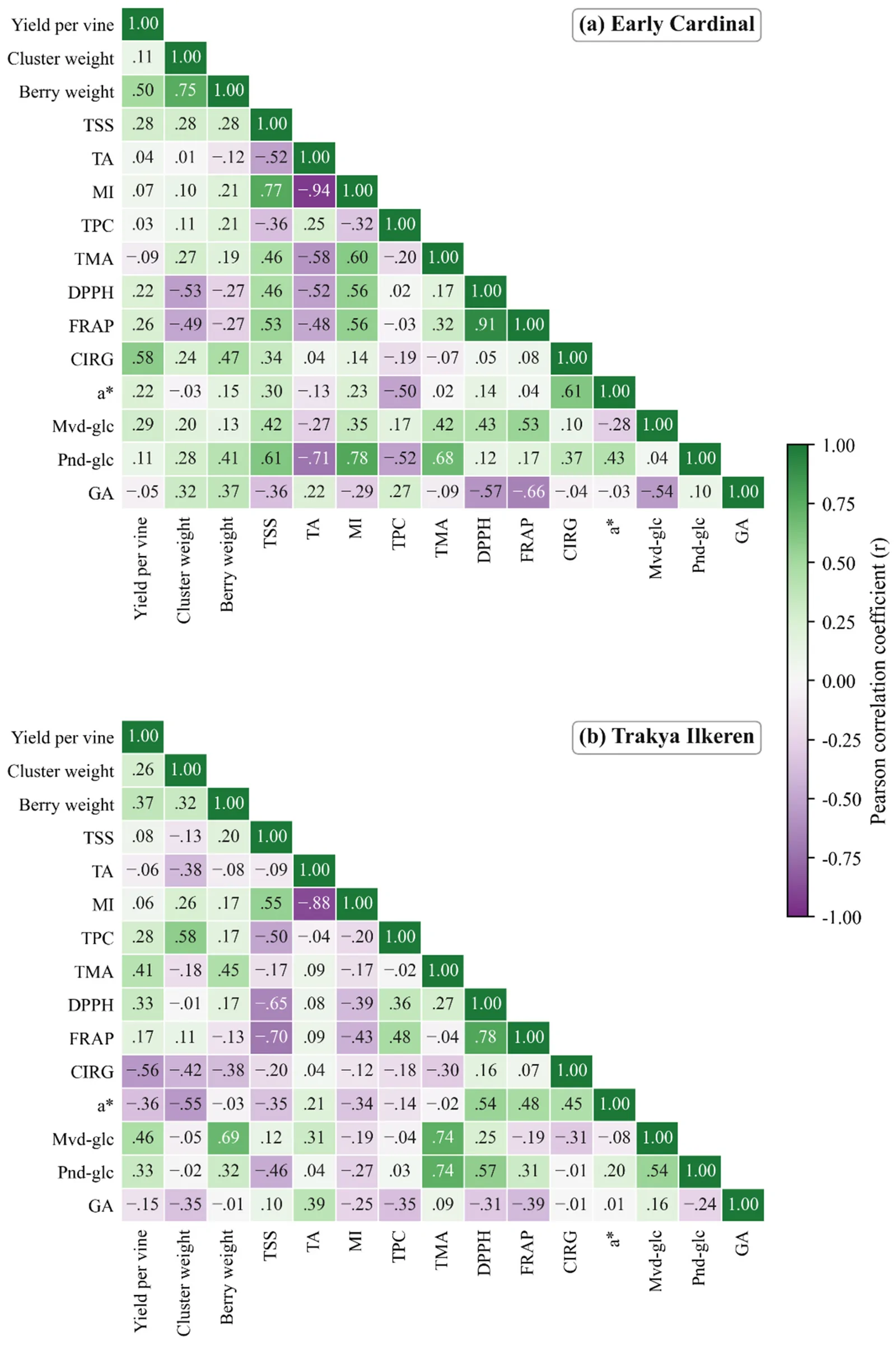
Lower-triangle Pearson correlation matrices among 15 key quality parameters in (**a**) ‘Early Cardinal’ and (**b**) ‘Trakya Ilkeren’. Colors range from purple (negative) to green (positive), and the coefficient (r) is printed in each cell. Correlations were computed on the complete replicate dataset (*n* = 15 per cultivar); |r| ≥ 0.51 corresponds to *p* < 0.05. TSS: Total soluble solids, TA: Titratable acidity, MI: Maturity index, TPC: total phenol content, TMA: total monomeric anthocyanins, DPPH: 1,1-diphenyl-2-picrylhydrazyl, FRAP: ferric reducing antioxidant power, a*: red/green scale, CIRG: Color Index for Red Grapes, Mvd-glc: Malvidin*-3-O-*glucoside, Pnd-glc: Peonidin*-3-O-*glucoside, GA: gallic acid.

**Table 1:** The effects on yield, cluster, and berry characteristics.

Cultivar	Treatments	Yield per Vine (kg)	Cluster Weight (g)	Cluster Length (cm)	Cluster Width (cm)	Berry Weight (g)	Berry Length (mm)	Berry Width (mm)
Early Cardinal	Control	8.66 ± 1.34	222.54 ± 8.37 c ^1^	22.87 ± 1.80 b	9.70 ± 0.21 c	4.71 ± 0.24 b	19.55 ± 0.38 b	19.28 ± 0.24 c
	Y1	7.68 ± 2.18	331.27 ± 10.25 a	23.97 ± 0.45 ab	12.25 ± 0.54 ab	5.48 ± 0.37 a	21.11 ± 0.35 a	21.40 ± 0.71 a
	Y2	9.66 ± 5.89	314.47 ± 29.11 a	25.58 ± 0.26 a	13.11 ± 0.89 a	5.13 ± 0.45 ab	20.93 ± 0.13 a	20.83 ± 0.48 ab
	Y3	7.93 ± 2.24	238.13 ± 12.73 c	23.33 ± 0.59 b	11.59 ± 0.97 b	4.81 ± 0.16 b	20.94 ± 0.28 a	20.49 ± 0.35 b
	Y4	10.46 ± 2.23	283.11 ± 14.50 b	25.56 ± 0.62 a	11.17 ± 0.89 bc	5.37 ± 0.18 a	20.57 ± 0.47 a	20.91 ± 0.33 ab
	LSD (5%)	NS	30.86	1.91	1.49	0.14	0.64	0.90
	*p*-value	0.8151	0.0001	0.0313	0.0074	0.0200	0.0032	0.0058
Trakya Ilkeren	Control	10.13 ± 2.25	296.50 ± 11.45 c	19.98 ± 0.80	11.61 ± 0.32 b	3.84 ± 0.18	17.77 ± 0.44	19.73 ± 0.59
	Y1	12.57 ± 2.94	332.83 ± 6.27 b	22.00 ± 1.60	11.43 ± 0.41 b	3.91 ± 0.35	17.36 ± 0.22	19.16 ± 0.38
	Y2	13.04 ± 1.73	397.01 ± 6.31 a	21.20 ± 1.17	12.38 ± 0.81 ab	4.13 ± 0.14	17.68 ± 0.60	19.78 ± 0.47
	Y3	13.05 ± 3.09	415.43 ± 25.58 a	21.64 ± 1.18	13.28 ± 0.56 a	4.01 ± 0.16	18.08 ± 0.46	19.65 ± 0.46
	Y4	13.46 ± 4.94	342.15 ± 24.47 b	21.93 ± 0.36	12.33 ± 0.52 ab	3.91 ± 0.24	17.48 ± 0.11	19.54 ± 0.30
	LSD (5%)	NS	35.26	NS	1.14	NS	NS	NS
	*p-*value	0.6778	0.0003	0.3238	0.0361	0.6486	0.3840	0.5798

^1^ Means followed by different lowercase letters in the same column are significantly different according to the LSD test at *p* ≤ 0.05. Letters are presented only for parameters for which the global ANOVA F-test was significant (*p* ≤ 0.05). NS: Non-significant. Results are the mean ± standard deviation of three independent experiments (*n* = 3). Y1: 0.5 kg ha^−1^, Y2: 1 kg ha^−1^, Y3: 2 kg ha^−1^, Y4: 4 kg ha^−1^ yeast doses.

**Table 2:** The effects on technological ripeness, sugars (g 100 g^−1^ FW), and organic acid (mg kg^−1^ FW) content of must.

Cultivar	Treatments	TSS (Brix)	pH	TA (g 100 mL^−1^)	MI	Glucose	Fructose	Sucrose	Tartaric Acid	Malic Acid	Citric Acid	Ascorbic Acid	Oxalic Acid	Fumaric Acid
Early Cardinal	Control	16.50 ± 0.20 ab ^1^	3.41 ± 0.03 bc	0.45 ± 0.03 a	36.64 ± 2.59 b	6.66 ± 0.15	6.38 ± 0.14 a	0.009 ± 0.0 c	4608.94 ± 63.77 b	1857.82 ± 130.18	108.88 ± 9.94 a	15.36 ± 1.07 bc	9.72 ± 2.21	4.21 ± 0.36 b
	Y1	16.07 ± 0.15 b	3.46 ± 0.01 ab	0.46 ± 0.01 a	35.01 ± 1.14 b	6.60 ± 0.26	6.43 ± 0.26 a	0.018 ± 0.0 a	4125.30 ± 179.81 c	1747.85 ± 61.60	53.57 ± 12.91 b	13.82 ± 1.90 c	9.63 ± 1.07	4.30 ± 0.40 b
	Y2	17.10 ± 0.61 a	3.41 ± 0.01 bc	0.45 ± 0.04 a	38.28 ± 2.96 b	6.65 ± 0.08	6.35 ± 0.08 ab	0.015 ± 0.0 b	4846.99 ± 237.67 ab	1823.53 ± 197.85	109.83 ± 11.31 a	17.14 ± 0.80 ab	11.83 ± 1.96	3.87 ± 0.17 b
	Y3	16.13 ± 0.40 b	3.36 ± 0.04 c	0.45 ± 0.02 a	35.53 ± 2.17 b	6.25 ± 0.27	5.95 ± 0.25 b	0.008 ± 0.0 c	4971.95 ± 106.13 a	1784.19 ± 105.03	95.97 ± 28.87 a	17.70 ± 1.29 a	11.04 ± 1.42	4.10 ± 0.16 b
	Y4	17.17 ± 0.64 a	3.47 ± 0.04 a	0.39 ± 0.01 b	43.69 ± 3.04 a	6.95 ± 0.19	6.67 ± 0.19 a	0.013 ± 0.0 b	4591.61 ± 167.76 b	2081.38 ± 54.89	58.69 ± 11.84 b	15.31 ± 0.60 bc	10.35 ± 1.35	5.82 ± 0.69 a
	LSD 5%	0.87	0.05	0.05	4.66	NS	0.41	0.003	301.37	NS	27.59	2.32	NS	0.61
	*p-*value	0.0500	0.0058	0.0471	0.0155	0.0559	0.0405	0.0004	0.0017	0.0772	0.0029	0.0288	0.4942	0.0006
Trakya Ilkeren	Control	17.43 ± 0.75	3.37 ± 0.04	0.55 ± 0.04 a	31.93 ± 3.73 bc	6.14 ± 0.38	5.77 ± 0.39	0.019 ± 0.0 a	4358.53 ± 122.19 b	3234.31 ± 195.69 cd	258.40 ± 24.12 a	3.71 ± 0.26	9.55 ± 0.51 c	5.21 ± 0.20 a
	Y1	16.37 ± 0.76	3.37 ± 0.03	0.49 ± 0.02 b	33.64 ± 2.13 ab	6.05 ± 0.06	5.66 ± 0.06	0.009 ± 0.0 c	4053.58 ± 113.12 d	3122.25 ± 166.37 d	129.15 ± 7.23 c	4.86 ± 0.18	3.83 ± 0.24 e	3.40 ± 0.50 c
	Y2	17.50 ± 0.62	3.38 ± 0.05	0.49 ± 0.01 b	35.95 ± 0.94 a	6.23 ± 0.13	5.85 ± 0.15	0.016 ± 0.0 ab	4229.30 ± 19.59 bc	3697.08 ± 444.16 ab	215.61 ± 20.01 b	5.12 ± 0.72	7.18 ± 0.35 d	5.41 ± 0.42 a
	Y3	16.63 ± 0.42	3.37 ± 0.02	0.51 ± 0.01 b	32.77 ± 1.29 b	5.97 ± 0.03	5.53 ± 0.04	0.014 ± 0.0 b	4144.42 ± 82.54 cd	3629.49 ± 101.68 bc	235.98 ± 21.30 ab	4.84 ± 0.39	11.51 ± 0.67 b	4.31 ± 0.07 b
	Y4	16.70 ± 0.30	3.34 ± 0.01	0.57 ± 0.01 a	29.33 ± 0.47 c	6.06 ± 0.02	5.68 ± 0.18	0.007 ± 0.0 c	4670.49 ± 37.92 a	4098.57 ± 290.22 a	107.86 ± 21.97 c	4.22 ± 0.89	12.94 ± 0.68 a	4.54 ± 0.17 b
	LSD 5%	NS	NS	0.031	4.464	NS	NS	0.004	161.11	453.43	40.60	NS	0.98	0.54
	*p-*value	0.0663	0.4412	0.0043	0.012	0.6635	0.4950	0.0005	0.0002	0.007	<0.0001	0.0642	<0.0001	0.0002

^1^ Means followed by different lowercase letters in the same column are significantly different according to the LSD test at *p* ≤ 0.05. Letters are presented only for parameters for which the global ANOVA F-test was significant (*p* ≤ 0.05). NS: Non-significant. Results are the mean ± standard deviation of three independent experiments (*n* = 3). Y1: 0.5 kg ha^−1^, Y2: 1 kg ha^−1^, Y3: 2 kg ha^−1^, Y4: 4 kg ha^−1^ yeast doses, TSS: total soluble solids, TA: titratable acidity, MI: maturity index, FW: Fresh weight.

**Table 3:** The effects on total phenolic content (mg GAE kg^−1^ FW) and individual polyphenols (mg kg^−1^ FW).

Cultivar	Treatments	TPC	GA	CGA	CFA	C	EC	ECG	RUT	Q3G	PC-A1	PC-A2	PC-B1	PC-C1
Early Cardinal	Control	3300.93 ± 97.04 c ^1^	10.92 ± 0.46 a	10.44 ± 0.12	59.86 ± 3.46 a	319.60 ± 27.98 a	170.68 ± 9.29	0.93 ± 0.08 c	82.62 ± 6.53 a	77.01 ± 3.47 a	213.22 ± 20.77	38.51 ± 6.84	34.75 ± 0.86 a	9.28 ± 0.57
	Y1	3866.36 ± 324.38 ab	8.69 ± 0.42 b	10.57 ± 0.73	25.08 ± 2.99 c	202.93 ± 39.1 c	110.96 ± 24.62	0.90 ± 0.20 c	51.52 ± 12.19 b	50.02 ± 10.16 c	147.62 ± 37.90	28.16 ± 7.61	21.61 ± 2.90 c	7.36 ± 4.29
	Y2	3717.51 ± 171.75 b	9.55 ± 0.72 b	10.62 ± 0.82	38.12 ± 7.74 b	294.00 ± 35.49 ab	159.77 ± 21.61	1.21 ± 0.03 ab	49.90 ± 10.08 b	55.14 ± 7.69 bc	228.21 ± 27.93	19.91 ± 2.34	30.92 ± 1.70 ab	7.64 ± 1.96
	Y3	4206.45 ± 158.09 a	9.36 ± 0.52 b	11.30 ± 1.82	42.26 ± 8.39 b	264.40 ± 46.94 abc	154.07 ± 24.16	1.37 ± 0.21 a	66.50 ± 5.41 ab	76.51 ± 7.36 a	183.84 ± 9.35	24.48 ± 6.15	29.65 ± 2.05 b	8.05 ± 0.65
	Y4	3598.86 ± 30.17 bc	9.24 ± 0.68 b	10.12 ± 0.11	41.41 ± 3.68 b	242.29 ± 35.39 bc	136.97 ± 16.20	1.00 ± 0.10 bc	71.52 ± 12.71 a	67.96 ± 11.02 ab	184.50 ± 33.97	26.44 ± 3.10	26.82 ± 3.54 b	7.80 ± 0.77
	LSD 5%	377.56	1.18	NS	9.42	74.41	NS	0.27	19.04	17.14	NS	NS	4.70	NS
	*p-*value	0.0059	0.0222	0.7042	0.0004	0.0472	0.0729	0.0162	0.0190	0.0200	0.0742	0.0511	0.0021	0.7646
Trakya Ilkeren	Control	2761.30 ± 87.80 c	9.54 ± 0.14	11.62 ± 1.50	21.26 ± 3.43	207.59 ± 45.63	136.54 ± 4.17 b	1.19 ± 0.27 b	46.41 ± 19.10	50.60 ± 10.75	151.94 ± 27.09 ab	23.68 ± 4.51	37.64 ± 1.32 b	7.98 ± 1.69
	Y1	3443.43 ± 80.65 b	10.01 ± 0.61	13.01 ± 0.48	23.55 ± 2.78	284.81 ± 45.53	166.03 ± 11.68 a	1.73 ± 0.30 a	37.19 ± 1.86	45.54 ± 1.72	191.67 ± 36.15 a	38.00 ± 11.39	49.46 ± 3.57 a	8.10 ± 1.40
	Y2	3382.96 ± 59.86 b	8.61 ± 0.26	12.64 ± 0.71	23.73 ± 3.39	191.46 ± 39.55	117.15 ± 7.62 b	0.76 ± 0.24 b	40.21 ± 0.64	44.10 ± 2.19	112.18 ± 14.70 b	19.45 ± 1.53	38.92 ± 3.91 b	7.57 ± 1.39
	Y3	3845.36 ± 211.13 a	10.14 ± 0.47	10.92 ± 1.40	18.96 ± 2.87	187.19 ± 64.34	126.24 ± 18.31 b	1.09 ± 0.15 b	37.86 ± 12.62	41.76 ± 6.00	115.10 ± 37.37 b	22.81 ± 6.66	37.01 ± 3.94 b	7.77 ± 1.32
	Y4	3891.02 ± 179.92 a	10.10 ± 1.28	11.89 ± 1.66	20.20 ± 4.43	235.98 ± 44.33	126.48 ± 17.62 b	0.86 ± 0.16 b	29.10 ± 9.04	45.75 ± 13.95	148.37 ± 33.35 ab	25.86 ± 9.22	39.43 ± 3.57 b	8.48 ± 1.83
	LSD 5%	223.74	NS	NS	NS	NS	28.86	0.474	NS	NS	51.97	NS	6.49	NS
	*p-*value	<0.0001	0.0585	0.3644	0.1698	0.1464	0.0325	0.0109	0.3875	0.6183	0.0417	0.1273	0.0120	0.9545

^1^ Means followed by different lowercase letters in the same column are significantly different according to the LSD test at *p* ≤ 0.05. Letters are presented only for parameters for which the global ANOVA F-test was significant (*p* ≤ 0.05). NS: Non-significant. Results are the mean ± standard deviation of three independent experiments (*n* = 3). Y1: 0.5 kg ha^−1^, Y2: 1 kg ha^−1^, Y3: 2 kg ha^−1^, Y4: 4 kg ha^−1^ yeast doses, TPC: total phenol content, GA: gallic acid, CGA: chlorogenic acid, CFA: caftaric acid, C: catechin, EC: epicatechin, ECG: epicatechin gallate, RUT: rutin, Q3G: quercetin-3-glucoside, PC-A1: procyanidin-A1, PC-A2: procyanidin-A2, PC-B1: procyanidin-B1, PC-C1: procyanidin-C1, FW: Fresh weight.

**Table 4:** The effects on total monomeric anthocyanins and skin color parameters.

Cultivar	Treatments	TMA (mg kg^−1^ FW)	L*	a*	b*	h*	C*	CIRG
Early Cardinal	Control	180.52 ± 27.13	32.08 ± 1.26	6.58 ± 0.62	−0.84 ± 0.41	−10.96 ± 3.12 a ^1^	7.09 ± 0.95	4.89 ± 0.30
	Y1	188.35 ± 22.06	31.27 ± 0.84	6.66 ± 0.90	−0.85 ± 0.58	−11.81 ± 3.29 a	6.10 ± 0.64	4.99 ± 0.28
	Y2	189.19 ± 8.70	32.31 ± 0.90	5.57 ± 0.47	−0.33 ± 0.18	−22.10 ± 2.99 b	5.81 ± 0.41	5.11 ± 0.12
	Y3	176.58 ± 16.96	32.55 ± 0.76	4.71 ± 0.89	−1.34 ± 0.74	−12.69 ± 1.55 a	5.19 ± 0.66	4.78 ± 0.20
	Y4	208.69 ± 7.48	33.41 ± 0.70	5.88 ± 0.73	−0.63 ± 0.71	−9.10 ± 2.06 a	6.87 ± 0.75	5.16 ± 0.08
	LSD 5%	NS	NS	NS	NS	5.11	NS	NS
	*p-*value	0.3645	0.2204	0.0819	0.3635	0.0029	0.0658	0.1671
Trakya Ilkeren	Control	230.58 ± 27.86	28.93 ± 1.41	3.34 ± 0.74	−1.17 ± 0.34 a	−29.21 ± 12.67 ab	3.90 ± 0.71	6.43 ± 0.26
	Y1	236.33 ± 19.13	26.89 ± 0.38	4.56 ± 0.37	−0.99 ± 0.12 a	−14.10 ± 2.31 a	4.76 ± 0.35	6.30 ± 0.29
	Y2	227.71 ± 28.11	27.33 ± 0.23	2.32 ± 0.66	−1.83 ± 0.20 b	−38.63 ± 8.36 b	3.09 ± 0.20	7.01 ± 0.17
	Y3	223.75 ± 41.54	27.62 ± 1.40	3.81 ± 0.79	−1.50 ± 0.48 ab	−19.30 ± 8.73 a	4.09 ± 0.61	6.37 ± 0.55
	Y4	232.51 ± 35.24	28.23 ± 2.07	3.37 ± 0.92	−0.97 ± 0.11 a	−21.72 ± 6.73 a	3.83 ± 0.84	6.44 ± 0.21
	LSD 5%	NS	NS	NS	0.54	15.18	NS	NS
	*p-*value	0.9901	0.2052	0.0748	0.0281	0.0399	0.1340	0.2020

^1^ Means followed by different lowercase letters in the same column are significantly different according to the LSD test at *p* ≤ 0.05. Letters are presented only for parameters for which the global ANOVA F-test was significant (*p* ≤ 0.05). NS: Non-significant. Results are the mean ± standard deviation of three independent experiments (*n* = 3). Y1: 0.5 kg ha^−1^, Y2: 1 kg ha^−1^, Y3: 2 kg ha^−1^, Y4: 4 kg ha^−1^ yeast doses, TMA: total monomeric anthocyanins, L*: lightness, a*: red/green scale, b*: yellow/blue scale, h*: hue angle (h values from 360–270° were considered negative), C*: chroma, CIRG: Color Index for Red Grapes, FW: Fresh weight.

**Table 5:** The Effects on individual anthocyanins (mg kg^−1^ FW).

Cultivar	Treatments	Mvd-glc	Pnd-glc	Ptd-glc	Dph-glc	Cyd-glc	Mvd*-p-*cm-glc	Mvd-ac-glc	Ptd*-p-*cm-glc	Pnd*-p-*cm-glc
Early Cardinal	Control	45.16 ± 2.86 c ^1^	87.87 ± 7.36 b	4.56 ± 0.18 b	3.48 ± 0.33	8.75 ± 0.89	10.97 ± 0.50 a	0.29 ± 0.16	1.59 ± 0.34 b	1.09 ± 0.22
	Y1	46.33 ± 2.94 bc	88.66 ± 1.98 b	4.24 ± 0.31 b	3.45 ± 0.95	9.03 ± 1.93	11.02 ± 1.07 a	0.28 ± 0.10	3.09 ± 0.78 a	1.20 ± 0.25
	Y2	60.45 ± 5.34 a	84.15 ± 2.72 b	5.92 ± 0.70 a	4.13 ± 0.37	8.19 ± 1.19	11.47 ± 0.70 a	0.24 ± 0.09	1.74 ± 0.22 b	1.01 ± 0.04
	Y3	55.35 ± 7.19 ab	74.02 ± 3.74 c	5.98 ± 0.22 a	3.81 ± 0.75	8.03 ± 0.67	9.35 ± 1.05 b	0.23 ± 0.10	1.73 ± 0.11 b	1.19 ± 0.14
	Y4	59.19 ± 4.39 a	104.44 ± 8.66 a	5.62 ± 0.27 a	4.53 ± 0.16	10.76 ± 0.52	12.40 ± 0.56 a	0.50 ± 0.21	1.65 ± 0.08 b	1.28 ± 0.17
	LSD (5%)	9.53	9.45	0.78	NS	NS	1.48	NS	0.78	NS
	*p-*value	0.0157	0.0010	0.0022	0.2790	0.1255	0.016	0.2423	0.0098	0.4690
Trakya Ilkeren	Control	127.53 ± 23.36	18.19 ± 5.25	10.29 ± 2.05	6.37 ± 1.15	1.26 ± 0.49	36.78 ± 4.86	3.73 ± 0.57	5.70 ± 0.45	2.06 ± 0.08
	Y1	122.57 ± 23.96	19.47 ± 2.60	10.85 ± 0.91	7.76 ± 0.42	1.52 ± 0.48	32.12 ± 7.08	3.38 ± 0.75	8.56 ± 2.04	2.04 ± 0.24
	Y2	131.22 ± 4.22	17.29 ± 2.78	10.86 ± 0.63	7.40 ± 0.35	1.25 ± 0.33	34.09 ± 0.83	3.13 ± 1.05	6.40 ± 0.57	2.25 ± 0.35
	Y3	123.24 ± 21.64	18.95 ± 1.72	10.21 ± 1.31	6.76 ± 0.69	1.53 ± 0.04	32.25 ± 6.31	3.15 ± 0.37	7.28 ± 3.01	1.86 ± 0.44
	Y4	125.70 ± 12.68	17.02 ± 2.88	11.70 ± 1.03	7.63 ± 1.04	1.42 ± 0.17	33.90 ± 3.69	3.93 ± 1.00	6.73 ± 0.56	2.14 ± 0.30
	LSD (5%)	NS	NS	NS	NS	NS	NS	NS	NS	NS
	*p-*value	0.9692	0.8378	0.5765	0.1982	0.7277	0.8382	0.6818	0.4530	0.6230

^1^ Means followed by different lowercase letters in the same column are significantly different according to the LSD test at *p* ≤ 0.05. Letters are presented only for parameters for which the global ANOVA F-test was significant (*p* ≤ 0.05). NS: Non-significant. Results are the mean ± standard deviation of three independent experiments (*n* = 3). Y1: 0.5 kg ha^−1^, Y2: 1 kg ha^−1^, Y3: 2 kg ha^−1^, Y4: 4 kg ha^−1^ yeast doses, Mvd-glc: malvidin*-3-O-*glucoside, Pnd-glc: peonidin*-3-O-*glucoside, Ptd-glc: petunidin*-3-O-*glucoside, Dph-glc: delphinidin*-3-O-*glucoside, Cyd-glc: cyanidin*-3-O-*glucoside, Mvd*-p-*cm-glc: malvidin*-3-O-*glucoside*-p-*coumaric, Mvd-ac-glc: malvidin*-3-O-*glucoside-acetate, Ptd*-p-*cm-glc: petunidin*-3-O-*glucoside-p-coumaric, Pnd*-p-*cm-glc: peonidin*-3-O-*glucoside*-p-*coumaric, FW: Fresh weight.

**Table 6:** The effects on antioxidant activity.

Cultivar	Treatments	DPPH (mg TE kg^−1^ FW)	FRAP (mg TE kg^−1^ FW)
Early Cardinal	Control	5340.82 ± 30.04 ab ^1^	4993.45 ± 462.35 b
	Y1	4200.93 ± 493.26 c	3126.03 ± 701.52 c
	Y2	5224.32 ± 281.94 b	5125.64 ± 210.03 ab
	Y3	5710.71 ± 298.39 ab	5332.74 ± 265.71 ab
	Y4	5872.19 ± 96.53 a	5767.21 ± 252.44 a
	LSD 5%	598.06	770.20
	*p-*value	0.0015	0.0004
Trakya Ilkeren	Control	6034.04 ± 431.36 bc	6022.34 ± 464.25 c
	Y1	6628.58 ± 133.79 a	7401.10 ± 672.54 ab
	Y2	5930.18 ± 240.29 c	5051.18 ± 513.26 d
	Y3	6366.10 ± 114.5 ab	7654.46 ± 569.46 a
	Y4	6281.63 ± 173.34 abc	6752.04 ± 615.17 bc
	LSD 5%	390.60	847.02
	*p-*value	0.0215	0.0006

^1^ Means followed by different lowercase letters in the same column are significantly different according to the LSD test at *p* ≤ 0.05. Letters are presented only for parameters for which the global ANOVA F-test was significant (*p* ≤ 0.05). Results are the mean ± standard deviation of three independent experiments (*n* = 3). Y1: 0.5 kg ha^−1^, Y2: 1 kg ha^−1^, Y3: 2 kg ha^−1^, Y4: 4 kg ha^−1^ yeast doses, DPPH: 1,1-diphenyl-2-picrylhydrazyl, FRAP: ferric reducing antioxidant power, FW: Fresh weight.

**Table 7:** Climatic parameters of the experimental region in the 2025 vintage.

Months	Avg. Temperature (°C)	Avg. Min. Temperature (°C)	Avg. Max. Temperature (°C)	Avg. Daily Radiation (Hour)	Total Rainfall (mm)	Relative Humidity (%)
January	12.47	6.68	18.65	5.69	0.30	55.51
February	8.96	4.21	14.59	7.24	29.20	53.34
March	16.51	9.92	24.13	7.57	12.30	51.06
April	17.73	11.68	24.88	6.65	28.20	56.18
May	22.16	16.75	29.14	9.04	37.10	62.25
June	25.88	21.21	31.61	11.04	1.70	67.64
July	29.52	24.97	35.55	10.34	0.60	69.87

## Data Availability

The raw data supporting the conclusions of this article will be made available by the authors on request.

## Supplementary Materials

The following supporting information can be downloaded at: https://www.mdpi.com/article/10.3390/molecules31193362/s1, Figure S1: Representative HPLC-PDA chromatograms of phenolic compounds at 280 nm (A), 320 nm (B), 355 nm (C), and 520 nm (D); Figure S2: Representative HPLC-RID chromatogram of sugar compounds; Figure S3: Representative HPLC-PDA chromatograms of organic acids at 210 nm (A) and ascorbic acid at 244 nm (B).

## Abbreviations

The following abbreviations are used in this manuscript:

Y1	0.5 kg ha^−1^ yeast treatment
Y2	1 kg ha^−1^ yeast treatment
Y3	2 kg ha^−1^ yeast treatment
Y4	4 kg ha^−1^ yeast treatment
C	Control
TPC	Total phenol content
TMA	Total monomeric anthocyanins
DPPH	1,1-diphenyl-2-picrylhydrazyl
FRAP	Ferric reducing antioxidant power
TA	Titratable acidity
MI	Maturity index
L*	Lightness
a*	Red/green scale
b*	Yellow/blue scale
h*	Hue angle
C*	Chroma
CIRG	Color Index for Red Grapes
Mvd-glc	Malvidin*-3-O-*glucoside
Pnd-glc	Peonidin*-3-O-*glucoside
Ptd-glc	Petunidin*-3-O-*glucoside
Dph-glc	Delphinidin*-3-O-*glucoside
Cyd-glc	Cyanidin*-3-O-*glucoside
Mvd*-p-*cm-glc	Malvidin*-3-O-*glucoside*-p-*coumaric
Ptd*-p-*cm-glc	Petunidin*-3-O-*glucoside*-p-*coumaric
Pnd*-p*-cm-glc	Peonidin*-3-O-*glucoside*-p-*coumaric
Mvd-ac-glc	Malvidin*-3-O-*glucoside-acetate
GA	Gallic acid
CGA	Chlorogenic acid
CFA	Caftaric acid
C	Catechin
EC	Epicatechin
ECG	Epicatechin gallate
RUT	Rutin
Q3G	Quercetin-3-glucoside
PC-A1	Procyanidin-A1
PC-A2	Procyanidin-A2
PC-B1	Procyanidin-B1
PC-C1	Procyanidin-C1
