# Supplementary material for: A Natural Approach to Improving Berry Quality in Table Grapes: Inactivated Yeast Extracts
Source: Molecules. 2026 Sep 22;31(19):3362. doi: 10.3390/molecules31193362 (PMC13665865; doi:10.3390/molecules31193362)
Supplement: Supplementary file 1 [file molecules-31-03362-s001.zip › molecules-4525337-supplementary.pdf]

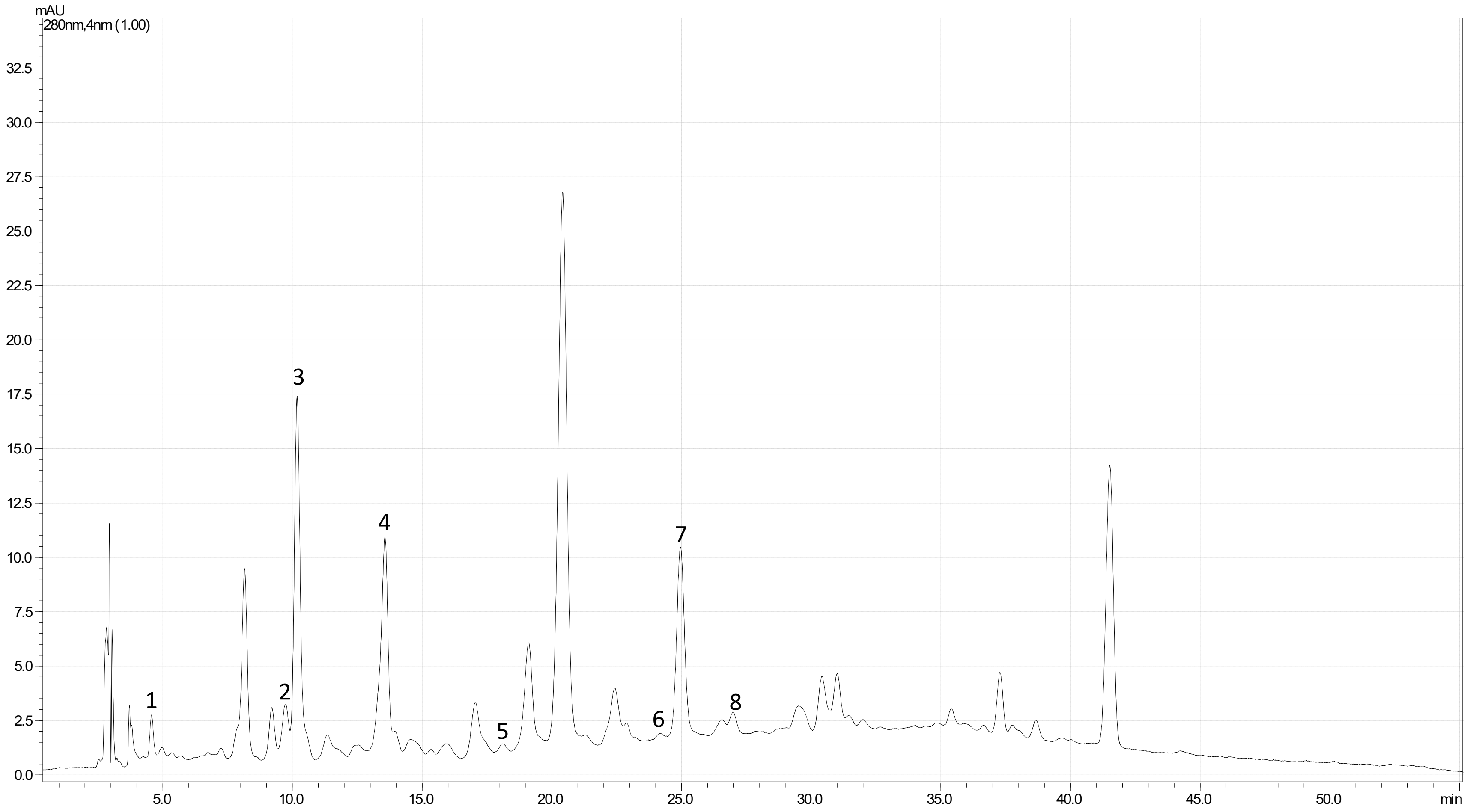

Figure S1A: The representative chromatogram (PDA) for phenolic compounds at 280 nm. 1: Gallic acid (GA), 2: Procyanidin-B1 (PC-B1), 3: Catechin (C), 4: Epicatechin (EC), 5: Procyanidin-C1 (PC-C1), 6: Epicatechin gallate (ECG), 7: Procyanidin-A1 (PC-A1), 8: Procyanidin-A2 (PC-A2).

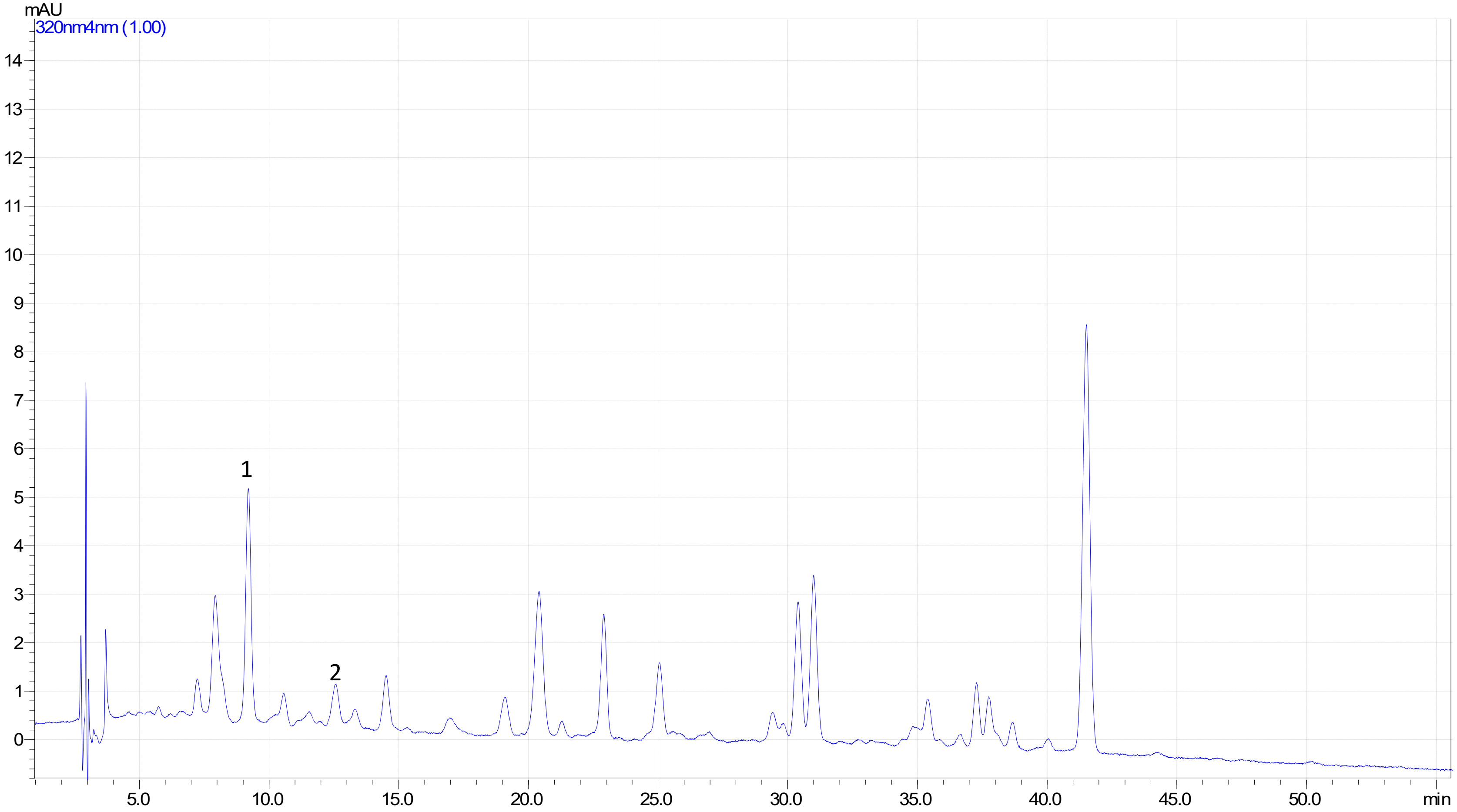

Figure S1B: The representative chromatogram (PDA) for phenolic compounds at 320 nm. 1: Caftaric acid (CFA), 2: Chlorogenic acid (CGA).

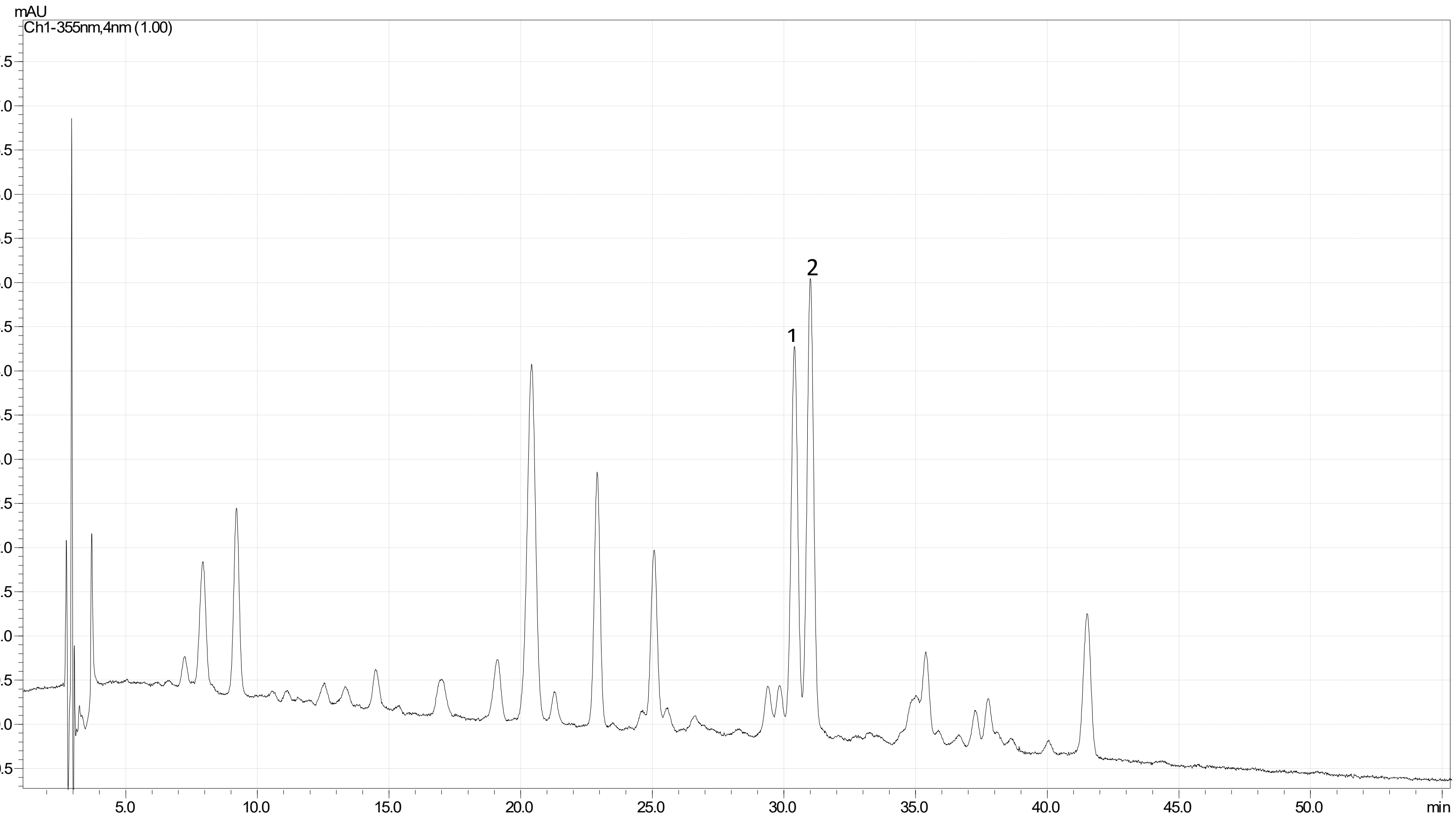

Figure S1C: The representative chromatogram (PDA) for phenolic compounds at 355 nm. 1: Rutin (RUT), 2: Quercetin-*3*-glucoside (Q3G)

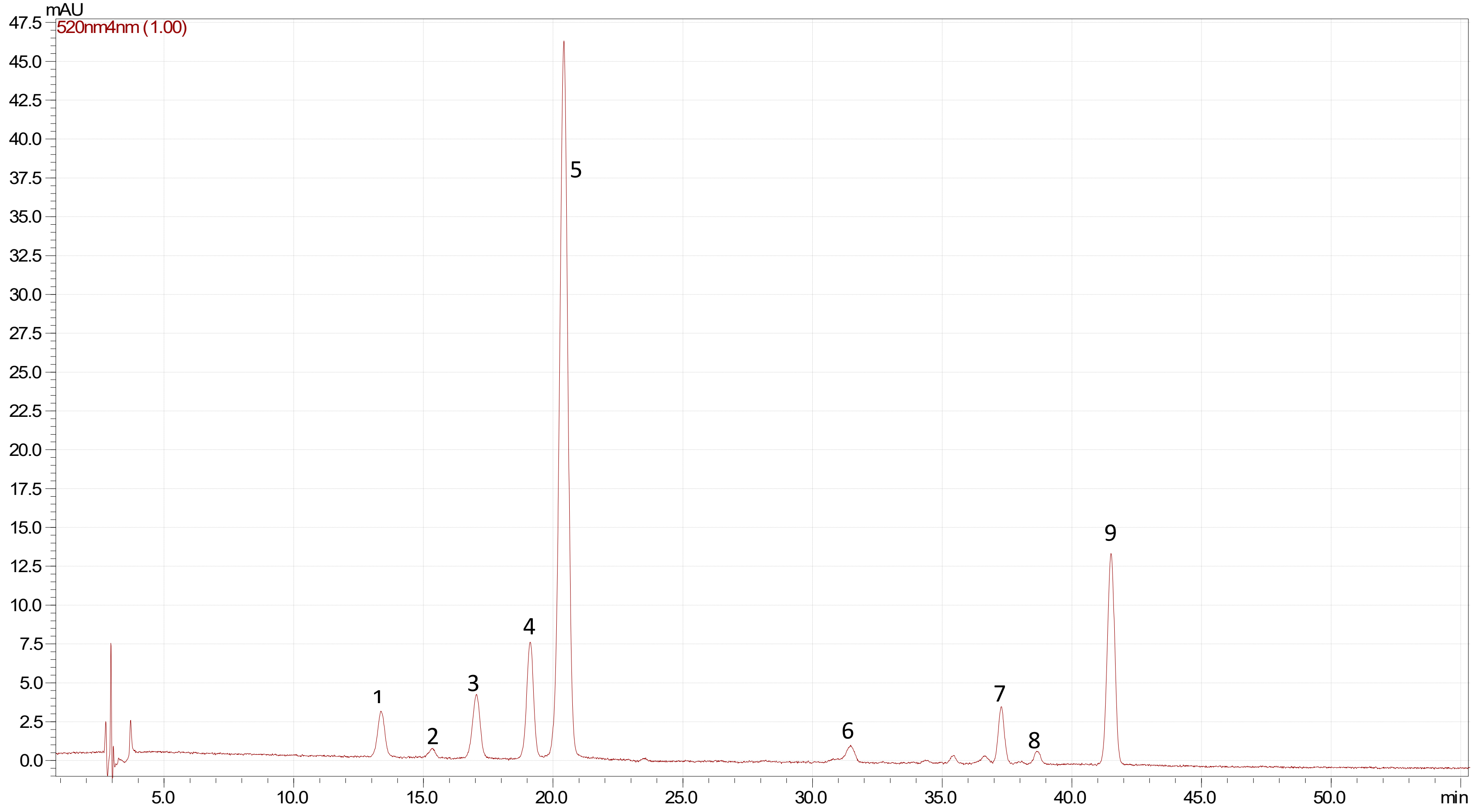

Figure S1D: The representative chromatogram (PDA) for phenolic compounds at 520 nm. 1: Delphinidin-*3*-*O*-glucoside (Dph-glc), 2: Cyanidin-*3*-*O*-glucoside (Cyd-glc), 3: Petunidin-*3*-*O*-glucoside (Ptd-glc), 4: Peonidin-*3*-*O*-glucoside (Pnd-glc), 5: Malvidin-*3*-*O*-glucoside (Mvd-glc), 6: Malvidin-*3*-*O*-glucoside-acetate (Mvd-ac-glc), 7: Petunidin-*3*-*O*-glucoside-*p*-coumaric (Ptd-*p*-cm-glc), 8: Peonidin-*3*-*O*-glucoside-*p*-coumaric (Pnd-*p*-cm-glc), 9: Malvidin-*3*-O-glucoside-*p*-coumaric) (Mvd-*p*-cm-glc)

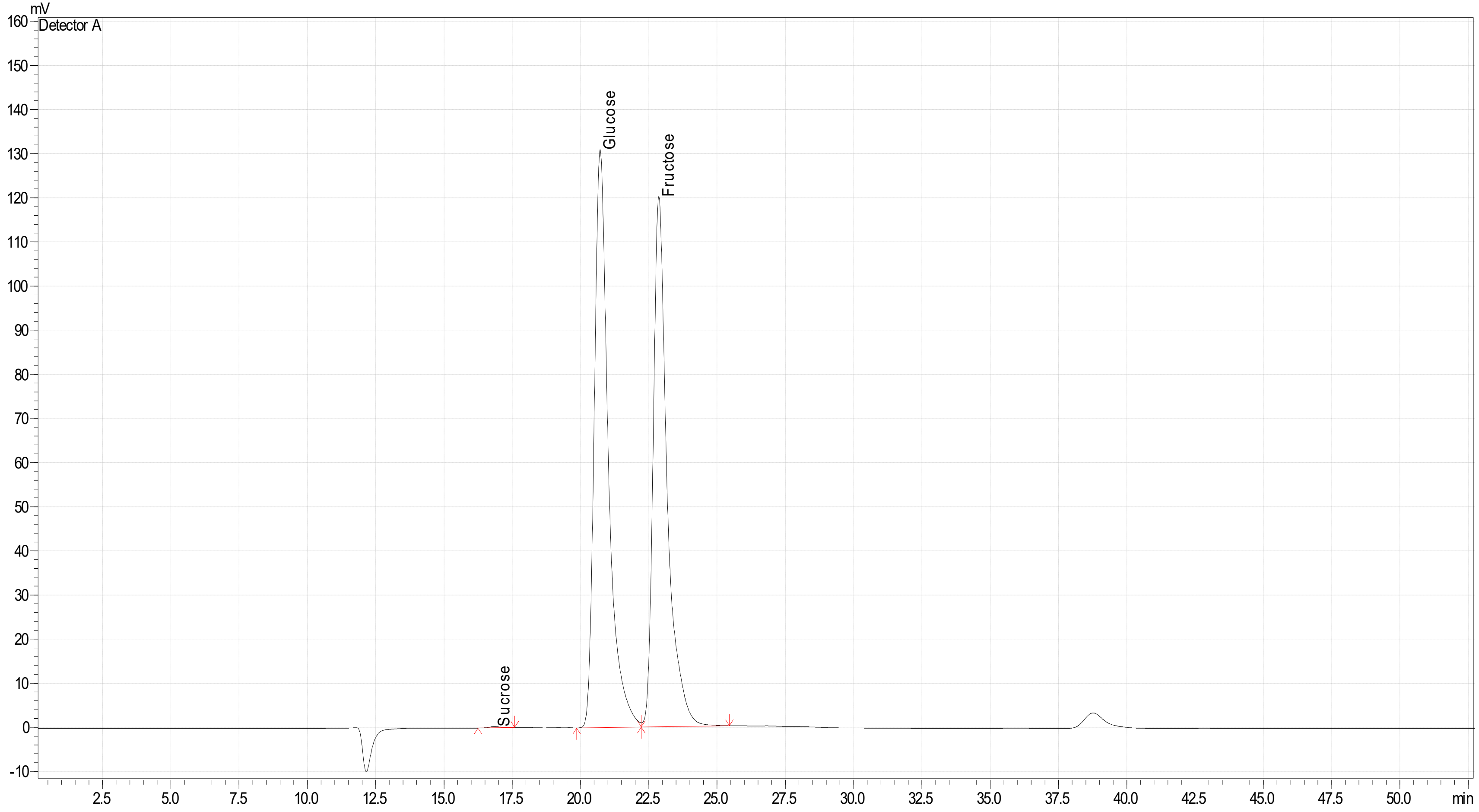

Figure S2: The representative chromatogram (RID) for sugar compounds

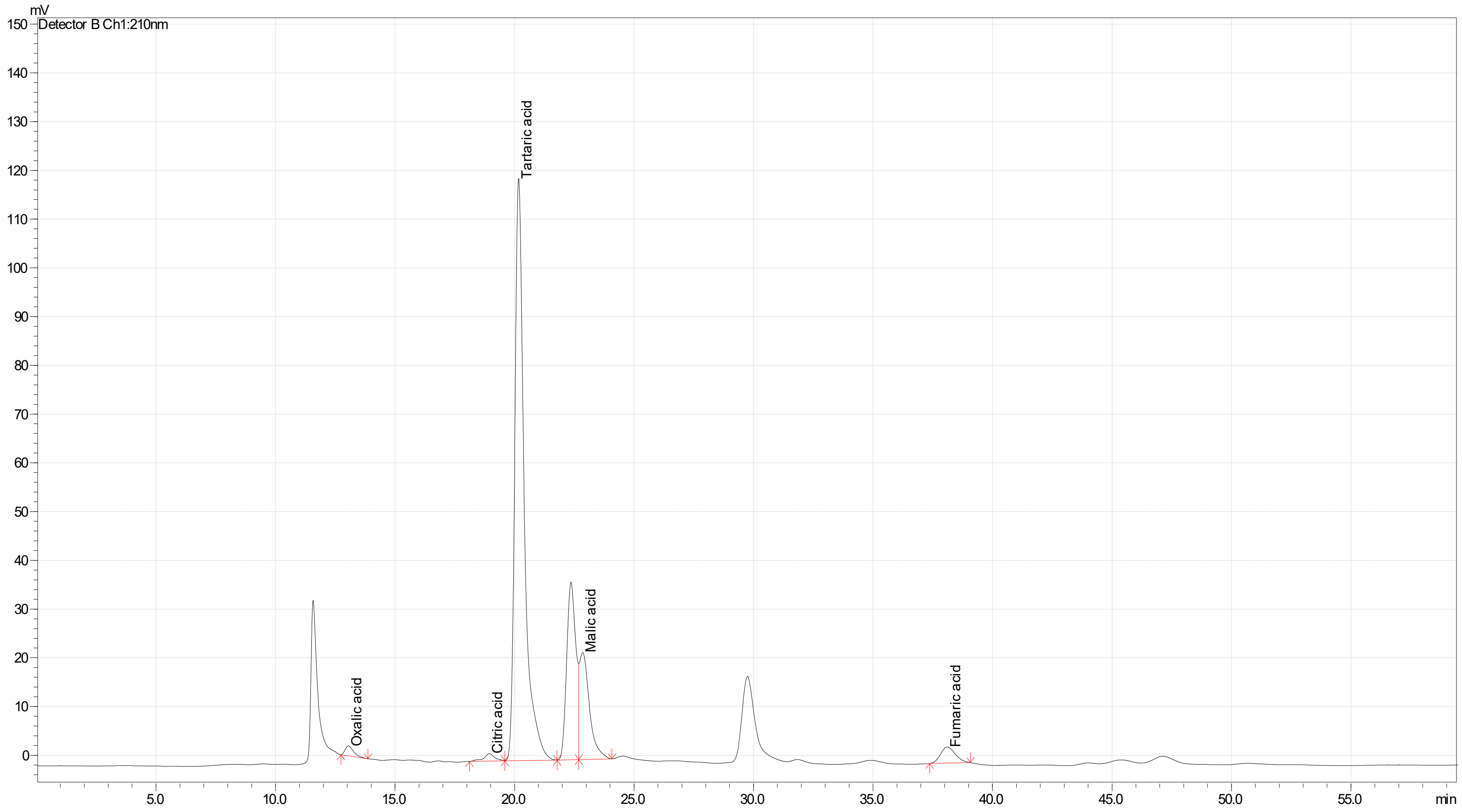

Figure S3A: The representative chromatogram (PDA) for organic acids at 210 nm

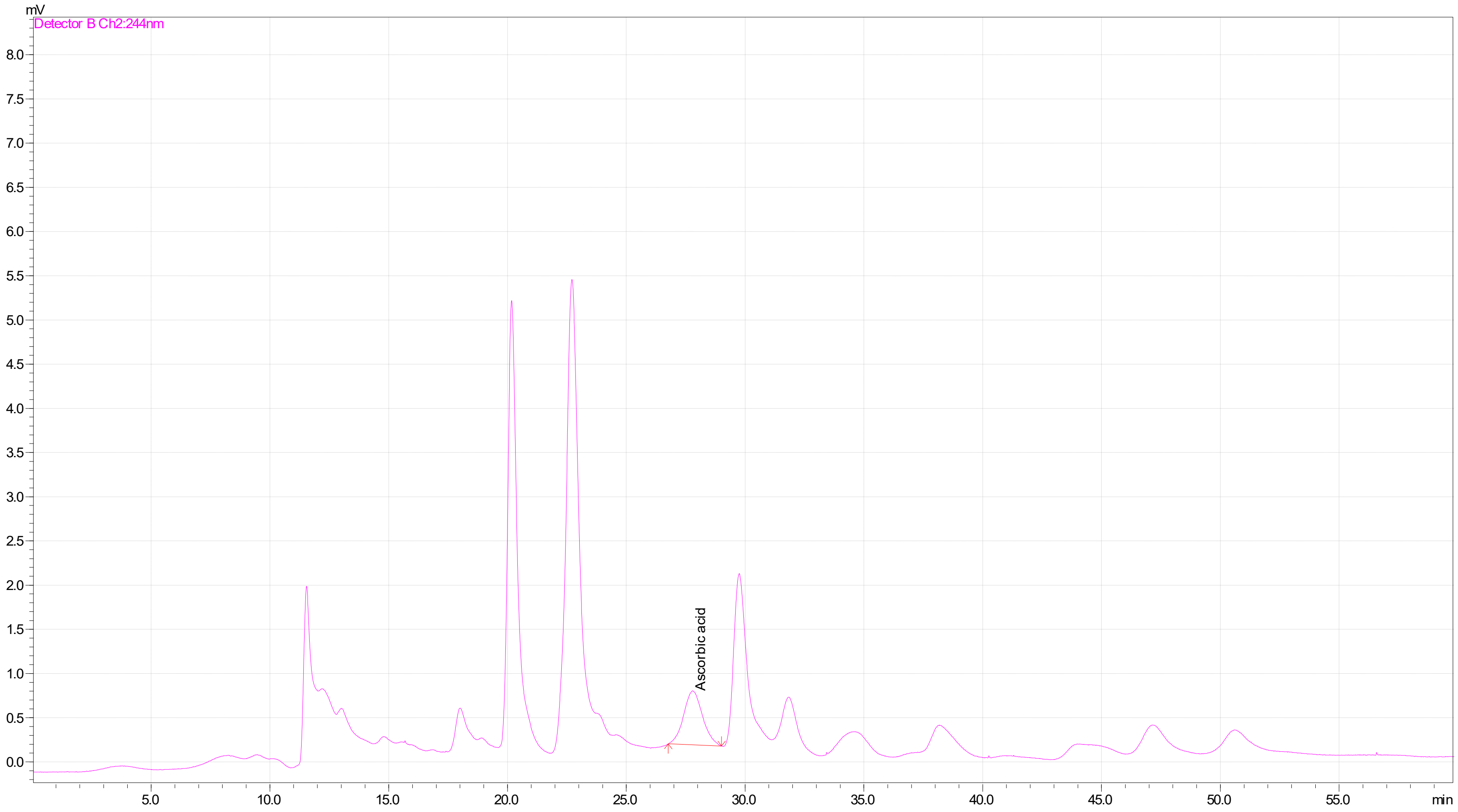

Figure S3B: The representative chromatogram (PDA) for ascorbic acid at 244 nm
